# In situ manipulation of van der Waals heterostructures for twistronics

**DOI:** 10.1126/sciadv.abd3655

**Published:** 2020-12-04

**Authors:** Yaping Yang, Jidong Li, Jun Yin, Shuigang Xu, Ciaran Mullan, Takashi Taniguchi, Kenji Watanabe, Andre K. Geim, Konstantin S. Novoselov, Artem Mishchenko

**Affiliations:** 1 School of Physics and Astronomy, University of Manchester, Oxford Road, Manchester M13 9PL, UK.; 2 National Graphene Institute, University of Manchester, Oxford Road, Manchester M13 9PL, UK.; 3 State Key Laboratory of Mechanics and Control of Mechanical Structures and MOE Key Laboratory for Intelligent Nano Materials and Devices, College of Aerospace Engineering, Nanjing University of Aeronautics and Astronautics, Nanjing 210016, China.; 4 National Institute for Materials Science, 1-1 Namiki, Tsukuba 305-0044, Japan.; 5 Centre for Advanced 2D Materials, National University of Singapore, 117546, Singapore.

## Abstract

In van der Waals heterostructures, electronic bands of two-dimensional (2D) materials, their nontrivial topology, and electron-electron interactions can be markedly changed by a moiré pattern induced by twist angles between different layers. This process is referred to as twistronics, where the tuning of twist angle can be realized through mechanical manipulation of 2D materials. Here, we demonstrate an experimental technique that can achieve in situ dynamical rotation and manipulation of 2D materials in van der Waals heterostructures. Using this technique, we fabricated heterostructures where graphene is perfectly aligned with both top and bottom encapsulating layers of hexagonal boron nitride. Our technique enables twisted 2D material systems in one single stack with dynamically tunable optical, mechanical, and electronic properties.

## Teaser

Rotation of 2D materials by polymer patch and gel stamp manipulator opens up a new strategy for twistronics.

## INTRODUCTION

Rotational misalignment caused by a twist angle between adjacent layers of two-dimensional (2D) materials results in symmetry breaking and strain effects, leading to enhanced or suppressed interlayer coupling and electronic band structure reconstruction (*1*–*4*). Moiré superlattices formed by rotational misalignment and lattice mismatch have been under intense scrutiny, and an abundance of new phenomena has been observed (*5*–*24*). Graphene placed on hexagonal boron nitride (hBN) represents a prototypical system of a twisted heterobilayer. In this system, when at a small twist angle, mono- or few-layer graphene in a quantizing magnetic field exhibits a fractal energy spectrum known as Hofstadter’s butterfly (*5*–*7*). This system has been reported to bear topological bands and strong correlations, exhibiting a number of fascinating many-body phenomena, such as correlated insulating states and superconductivity reported in trilayer graphene on hBN (*8*, *9*), and fractional Chern insulators observed in bilayer graphene on hBN (*10*). In twisted homobilayers, strongly correlated phenomena including correlated insulating states, unconventional superconductivity, and ferromagnetism have been observed in twisted bilayer graphene (*11*–*14*) and twisted bilayer-bilayer graphene systems (*15*–*18*). A variety of other systems, such as twisted transition metal dichalcogenide layers (*19*–*22*), graphene/WS_2_ bilayer (*23*), and twisted bilayer graphene aligned to hBN (*24*), have also manifested many intriguing phenomena.

In twistronics, accurate positioning, rotation, and manipulation of 2D materials are needed to fabricate a system with desired twist angles. To this end, several techniques have been developed, including optical alignment of crystal edges (*7*, *25*), tear-and-stack technique for twisted homobilayers (*26*), and in situ rotation mediated by atomic force microscopy (AFM) tips (*27*). Here, we present a new experimental strategy to dynamically manipulate layered heterostructures in situ with precise control, allowing investigation of optical, mechanical, and electronic properties of a system with tunable twist angles between individual layers.

## RESULTS

**Fig. 1:**
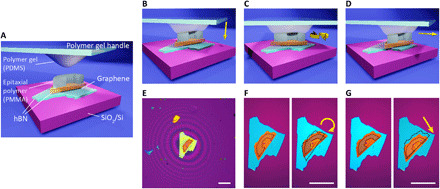
In situ manipulation of van der Waals heterostructures. (**A**) Schematic of polymer (PMMA)–mediated in situ manipulation technique. (**B**) Schematic of the polymer gel handle (PDMS handle) touching PMMA. (**C**) Schematic of rotating a 2D material stack. (**D**) Schematic of sliding a 2D material stack. (**E**) Optical image of the stack covered by a polymer (PMMA) patch in contact with the polymer gel (PDMS). The interference rings show the proximity of the PDMS hemisphere to the substrate. (**F**) Optical images of the stack before (left) and after (right) rotation. The yellow arrow shows the rotation direction. (**G**) Optical images of the stack before (left) and after (right) translation manipulation. The yellow arrow shows the translation direction. The dashed lines in the right panels of (F) and (G) indicate the original position of the graphene and top hBN. Scale bars, 40 μm.

For our in situ twistronics technique, we use a glass slide with a droplet of polydimethylsiloxane (PDMS) as a manipulator, which is cured and naturally shaped into a hemisphere geometry. For a carefully fabricated PDMS hemisphere, the contact area between the manipulator and a 2D crystal can be as small as a few tens of micrometers (*26*, *28*), which depends on the hemisphere radius and is highly sensitive to the contact force, making it difficult to precisely control the target flake. To solve this problem, we developed a critical step, where we intentionally deposited an epitaxial polymethyl methacrylate (PMMA) patch on top of a target flake through a standard electron beam lithography (EBL) process. The concept is illustrated in Fig. 1. The epitaxial PMMA patch can be designed into an arbitrary shape that fits the target flake, and its thickness is normally a few hundred nanometers, which can be further increased by spin coating double layers of PMMA or by reducing the spinning speed, while the height of the PDMS manipulator can be adjusted with a high accuracy in most of the micromanipulator-based transfer systems. Thereby, the contact area between the PDMS hemisphere and the flake can be precisely limited to the area of the epitaxial PMMA patch without PDMS touching beyond the target flake at a much larger contact force threshold.

This strategy facilitates the accurate manipulation of the target flake. Figure 1 (B to D) shows the cartoon schematics of how the in situ manipulation of heterostructures works. To manipulate the target flake of the hBN/graphene/hBN heterostructure, first, by lowering down the polymer gel handle, PDMS hemisphere is brought in contact with the PMMA patch, which is prepatterned onto top hBN. When they touch, there is a color change in the PMMA patch, which can be easily distinguished under an optical microscope (see fig. S1). Figure 1E shows the top view when the PDMS hemisphere touches the PMMA patch, where the interference rings imply that the PDMS hemisphere is in the proximity of the flake without touching it. In incommensurate state, the top hBN and the underneath graphene can both slide and rotate freely on the surface of the bottom hBN under the control of the PDMS hemisphere (Fig. 1, C, D, F, and G, and movies S1 and S2). Figure 1 (F and G) shows that the PMMA patch is in good adhesion to the top hBN and does not delaminate during the manipulation process. We discuss, in detail, the motion mechanism of incommensurately stacked 2D materials in section S2. Briefly, sliding of the crystallites is governed by the friction forces at graphene/top hBN and graphene/bottom hBN interfaces. In incommensurate state, kinetic friction forces at the two interfaces are markedly reduced, which is the so-called superlubricity (*29*, *30*), and could be slightly different from each other depending on the twist angles θ_t_ and θ_b_, as well as the interface conditions; therefore, the graphene can rotate or slide with the movement of the top hBN.

The manipulation technique presented here enables continuous modification of the twist angle between the layers even after the heterostructure is already assembled. To perform in situ optical measurements such as Raman spectroscopy after each manipulation step, the PMMA patch was intentionally designed not to cover the graphene so that it can give a strong enough signal (Fig. 1, F and G). Compared with previous in situ rotation technique mediated by an AFM tip (*27*), our manipulation technique is convenient and reproducible since the PMMA patch can be easily washed away by acetone and repatterned by EBL. In addition, our technique can manipulate flakes regardless of their thickness, whereas an AFM tip might destroy thin flakes.

**Fig. 2:**
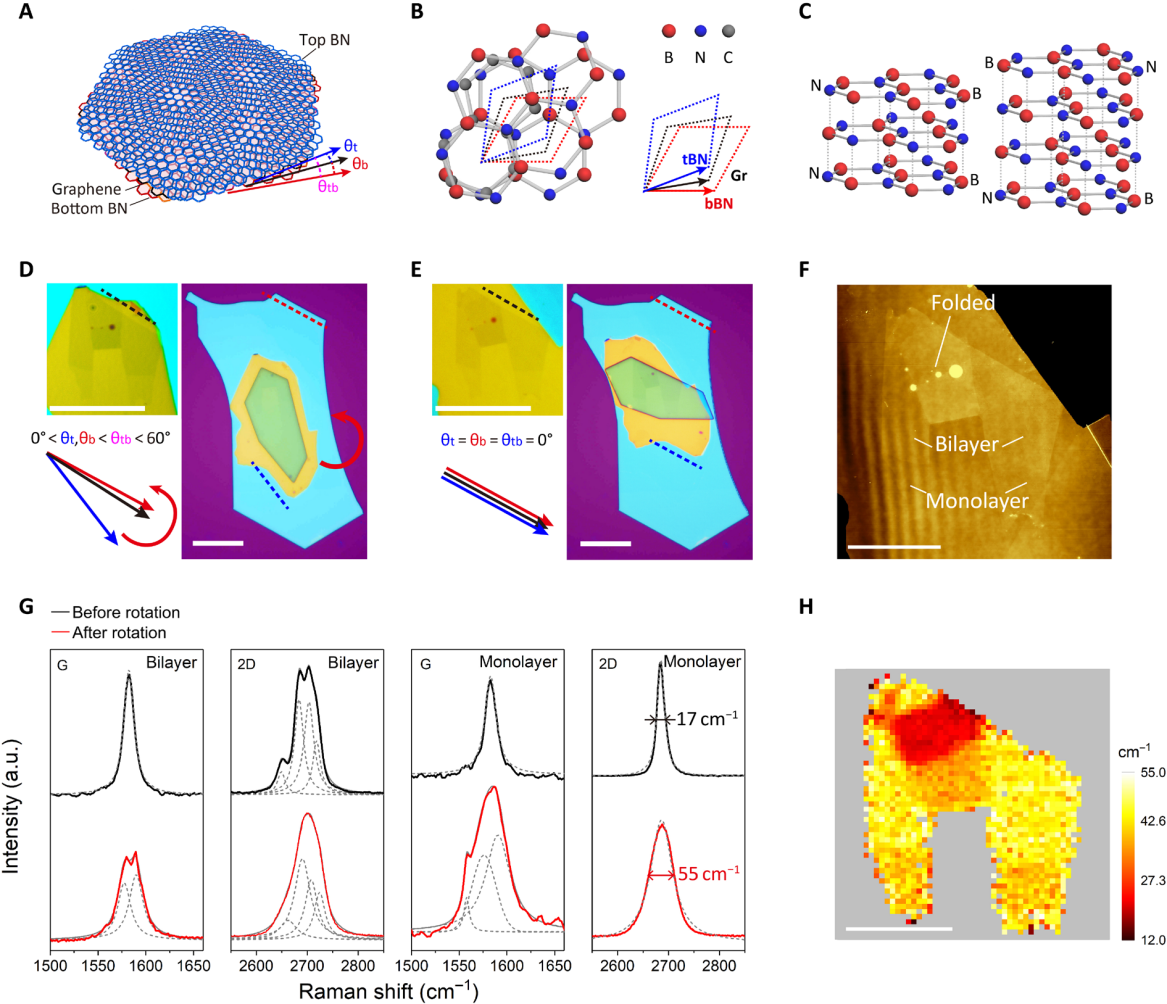
Encapsulated graphene perfectly aligned to both the top and bottom hBN using in situ rotation technique. (**A**) Schematic of graphene encapsulated by hBN, with twist angles θ_t_, θ_b_, and θ_tb_ between the layers. (**B**) Lattice structure of graphene encapsulated by hBN and the corresponding lattice vectors of each layer. (**C**) Atomic structure of hBN, with odd (left) and even (right) numbers of layers. (**D** and **E**) Optical images of the stack, before (D) and after (E) rotation, which belong to the scenarios in fig. S3A (before rotation) and fig. S3E (after rotation). The top left panels increase the contrast to show the position of graphene. The bottom left panels show the relative crystal orientations of each layer. The red arc arrow highlights the rotation direction. The dashed lines indicate the crystal edges of graphene and hBN layers, which were aligned after the PMMA patch delaminated from the top hBN, as shown in (E). Scale bars, 20 μm. (**F** and **H**) AFM topography and 2D bandwidth Raman map of the stack after rotation, respectively. Scale bars, 10 μm. (**G**) Raman spectra of graphene at the monolayer and bilayer regions before and after rotation. a.u., arbitrary units.

To demonstrate the potential of the manipulation technique in twistronics, we fabricated another hBN/graphene/hBN heterostructure (sample 1) where the graphene layer was aligned to both the top and bottom hBN layers. We first assembled the heterostructure intentionally with 0 < θ_t_, θ_b_ < θ_tb_ < 60° (Fig. 2D), where θ_t_, θ_b_, and θ_tb_ are twist angles between graphene and top hBN, graphene and bottom hBN, and top hBN and bottom hBN, as shown in Fig. 2 (A and B). Then, we used our technique to rotate the top hBN layer, by making sure that the rotating PMMA patch is patterned within the top layer (Fig. 2D). The twist angles θ_t_ and θ_b_ varied simultaneously as the rotation progressed until either of them reached 0° where the graphene layer was locked to one of the hBN layers, that is, the graphene/hBN interface went through a transition from an incommensurate to a commensurate state (*31*, *32*). At a certain moment, the smooth rotation stacks and the PMMA delaminates from the top hBN layer (Fig. 2E). We associate these conditions with θ_t_ and θ_b_ both being ≈0°, indicating that graphene is aligned to both hBN layers. At this stage, the two hBN layers are aligned to each other as well, with θ_tb_ also reaching 0°, as shown in Fig. 2E. The delamination of PMMA after all the 2D layers are locked to each other indicates that the interaction between the 2D materials at the commensurate state is stronger than that between the PMMA and the top 2D crystal (see section S2). Such a self-locking mechanism is inevitable in commensurately stacked 2D materials and hinders further rotation and tuning of twist angles (*33*). If one imposes a larger external driving force to further rotate or move the crystals after locking, it might risk destroying the 2D crystals (see example shown in fig. S2, where we slightly etched the top hBN and then deposited the PMMA patch so that it will not delaminate from the top hBN). For other 2D systems where the commensurate state does not happen (see fig. S6), the twist angle can be reversibly tuned even at marginal values.

In principle, there are two types of alignment in a doubly aligned hBN/graphene/hBN heterostructure, that is, θ_t_ = θ_b_ = θ_tb_ = 0° and θ_t_ = 0°, θ_b_ = θ_tb_ = 60°. The state of the resulting stack is determined by the initial settings of θ_t_ and θ_b_ and the rotation direction. Scenarios for various θ_t_ and θ_b_ are presented in fig. S3, where the cases in fig. S3A (initial alignment) and fig. S3E (final alignment) match Fig. 2D and Fig. 2E. The initial settings of the twist angles rely on the known crystal orientation of graphene and hBN layers, which is normally distinguished by straight long edges of the flake. Since hBN crystals are threefold rotationally symmetric, using hBN flakes from the same exfoliated crystal can help to secure the crystal orientations. However, for hBN, the symmetry between the top and bottom atomic layers depends on whether the number of layers in hBN is odd or even (Fig. 2C). This ambiguity can, in principle, be circumvented by using the same surface, either top or bottom, of the original hBN crystal for alignment. To demonstrate how this can be done, we fabricated sample 2 with θ_t_ = 0°, θ_b_ = θ_tb_ = 60° in the final stack (see section S2 and figs. S4 and S5).

## DISCUSSION

To confirm the alignment of graphene to both hBN layers, we carried out Raman characterization, as shown in Fig. 2 (G and H). The graphene layer in sample 1 originally contained monolayer and bilayer regions (Fig. 2F). For the monolayer region, the full width at half maximum of 2D peak (FWHM_2D_) increases from 17 to 55 cm^−1^ after rotation; G peak width also broadens with the emergence of lower-frequency components (Fig. 2G). These results are consistent with previous reports (*34*, *35*). The broadening of 2D peak by near 40 cm^−1^ and the appearance of lower-frequency components in G peak signify near-perfect alignment between the hBN and graphene crystals and arise from graphene coupling to the moiré potentials from both top and bottom hBN crystals (*27*, *35*). For the bilayer region, the splitting of 2D peak is strongly enhanced when graphene is misaligned to both hBN layers, whereas after rotation, these components broaden and shift toward each other, resulting in a prominent change in the line shape of 2D peak. In contrast, G peak splits into two distinct components after rotation. These results for doubly aligned bilayer graphene have not been reported before, and we ascribe the change in Raman spectra to the periodic strain field induced by moiré patterns (see section S3). Figure 2H shows the 2D peak width Raman map of the graphene layer after rotation. It clearly demonstrates the homogeneous distribution of FWHM_2D_ among different regions of the graphene layer, indicating a spatially uniform twist angle in the stack. The AFM topography (Fig. 2F) here shows that the rotation process did not damage or crease the graphene layer.

**Fig. 3:**
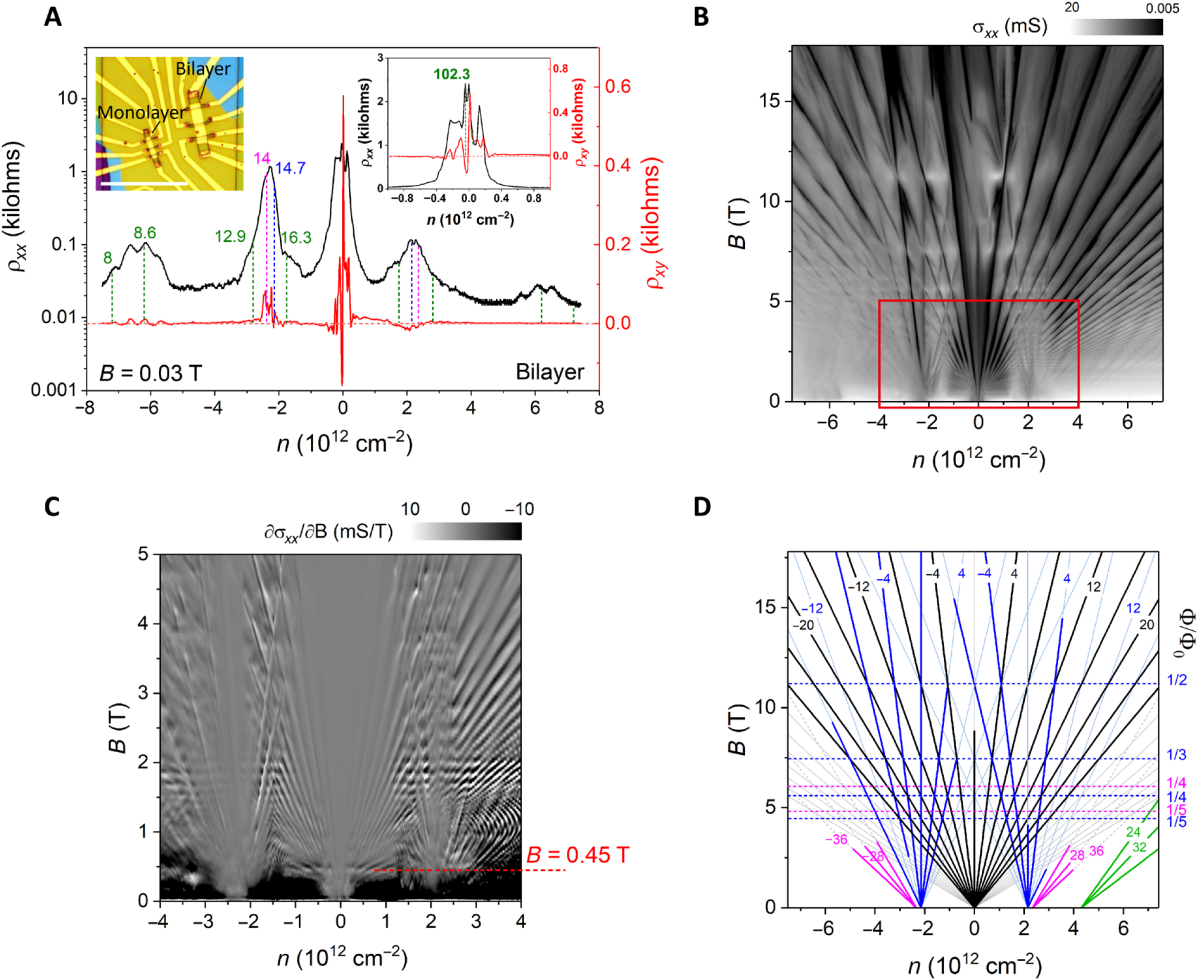
Hofstadter’s butterfly and Brown-Zak oscillations in bilayer graphene double moiré superlattices. (**A**) ρ*_xx_* and ρ*_xy_* as a function of *n*. *T* = 0.3 K, *B* = 0.03 T. The blue, magenta, and green dashed lines and numbers mark *n*_s1_, *n*_s2_, and *n*_sm_, with moiré wavelengths λ_s1_, λ_s2_, and λ_sm_, respectively. Left inset shows device micrograph. Scale bar, 20 μm. Right inset highlights the low carrier density region of (A). (**B**) Map σ*_xx_*(*n*,*B*) measured at *T* = 0.3 K. (**C**) Map ∂σ*_xx_*/∂*B*(*n*,*B*) highlighting Brown-Zak (BZ) oscillations in part (red rectangle) of (B). The feature at *B* = 0.45 T originates from the BZ state of the super-moiré pattern with λ_sm_ ≈ 102.3 nm. (**D**) Wannier diagram labeling the quantum Hall states identified in (B). The solid lines show quantum oscillations emerging from PDP and SDPs, with ν = ±4, ±8, ±12, ... for PDP (black), *t* = ±4, ±8, ±12, ... for SDP *n*_s1_ (blue), *t* = ±20, ±28, ±36 for SDP *n*_s2_ (magenta), and *s* = 2, *t* = 24, 32, 44 for SDP *n*_s1_ (green). The horizontal dashed lines and numbers on the right show the most prominent BZ oscillations of SDPs *n*_s1_ (blue) and *n*_s2_ (magenta), with different values of *p*/*q* for ϕ = (*p*/*q*) ϕ_0_.

To further quantify the existence of two moiré superlattices at both sides of the graphene layer in this heterostructure, we made it into a device and investigated its transport properties. We will now focus on our bilayer device (Fig. 3). Figure 3A shows the longitudinal resistivity ρ*_xx_* and transverse resistivity ρ*_xy_* as a function of charge carrier density *n* at nonquantizing magnetic field of *B* = 0.03 T. We observed both broadened resistivity peak at the primary Dirac point (PDP) and satellite resistivity peaks at finite densities situated symmetrically with respect to PDP. The Hall resistivity ρ*_xy_* near each satellite peak changes sign, suggesting that they are moiré-induced secondary Dirac points (SDPs).

In quantizing magnetic fields, the presence of moiré potential splits the Landau levels into a fractal structure: the Hofstadter minibands separated by a hierarchy of self-similar minigaps, to which the corresponding densities follow linear trajectories according to the Diophantine equation: *n*/*n_0_* = *t* (ϕ/ϕ_0_) + *s*, where *s* and *t* are integers denoting the superlattice miniband filling index and quantized Hall conductivity of the gapped state, respectively; ϕ_0_ is the magnetic flux quantum; and *n_0_* is the total number of electron states per area of a completely filled Bloch band (*36*). Existence of two moiré patterns should result in two sets of these self-similar bands. Figure 3D is a simplified Wannier diagram showing the positions of the most prominent σ*_xx_* zeroes in the measured Landau fan diagram (Fig. 3B). We find that the Landau fans originate from PDP at *n* = 0 and from the two sets of SDPs at *n*_s1_ = ±2.15 × 10^12^ cm^−2^ and *n*_s2_ = ±2.34 × 10^12^ cm^−2^ (corresponding to the moiré wavelengths of λ_s1_ = 14.7 nm and λ_s2_ = 14.0 nm, respectively). To identify the two SDPs more clearly, we plot ∂σ/∂*n* versus *B* and *n* in fig. S9 (A, C, and D).

In the fractal superlattice spectra, the self-similar minigaps occur at ϕ = ϕ_0_*p*/*q*, where ϕ = *BA* is the magnetic flux per superlattice unit cell, *A* is the superlattice unit cell area, ϕ_0_ is the magnetic flux quantum, and *p* and *q* are co-prime integers. The strongest minigaps arise at ϕ = ϕ_0_/*q*, resulting in Brown-Zak (BZ) magneto-oscillations (*37*) with the periodicity of 1/*B* = *A*/ϕ_0_. These minigaps also correspond to the intersections between the central and satellite fans in the Landau fan diagram (*5*, *6*). We observe BZ oscillations (horizontal streaks visible in Fig. 3B) belonging to the two sets of satellite fans, as shown by the horizontal blue and magenta dashed lines in Fig. 3D, which provides independent confirmation of the two distinct moiré periods. BZ oscillations are more clearly seen in ∂σ/∂*B*(*B*,*n*) maps in fig. S9 (B, E, and F), in which we observed other *p*/*q* fractions. High temperature suppresses cyclotron oscillations, making the BZ oscillations more visible (fig. S10). At electron doping where BZ oscillations are more pronounced (*37*), two sets of maxima, corresponding to the two periodicities, 15.2 and 14.7 nm, are clearly seen (fig. S10, B and D). This independently verifies the existence of two moiré superlattices with different wavelengths at both sides of the graphene layer.

These coexisting moiré patterns with different wavelengths should, in principle, interfere, resulting in a second-order (composite) moiré pattern, as reported in experimental (*38*–*40*) and theoretical studies (*41*, *42*). From the two SDPs, we calculated the corresponding twist angles to be 0.24° and 0.38° (see Materials and Methods). Using the method described in (*38*, *39*), we obtained six possible composite moiré wavelengths (λ_sm_; see section S4) and observed satellite peaks in ρ*_xx_* near most of the corresponding carrier densities *n*_sm_ required to reach the first Brillouin zone edge of the six possible composite moiré patterns, which are marked by green dashed lines and numbers in Fig. 3A. The *n*_sm_ of the largest λ_sm_ ≈ 102.3 nm is in the range of ±0.04 × 10^12^ to ±0.05 × 10^12^ cm^−2^ (right inset of Fig. 3A). Notably, in the plot of ∂σ_xx_/∂*B*(*n*,*B*) within |*n*| < ±2 × 10^12^ cm^−2^, we observe a prominent horizontal streak at *B* = 0.45 T (Fig. 3C), which perfectly matches the magnetic field of the first-order magnetic Bloch state originating from the composite moiré pattern when *BA* = ϕ_0_ (where *q* = 1). The results for the bilayer graphene region discussed above are similar to what was found in the monolayer graphene region (figs. S11 to S13).

The manipulation technique presented here proves success in making the hBN/graphene/hBN heterostructures with perfect alignment between graphene and the two hBN layers with a high twist angle homogeneity. In contrast, optical alignment of crystal edges or heating of the final stack cannot consistently guarantee perfect alignment with the twist angle of <1° in a single stack (*5*–*7*, *10*, *38*, *40*). Among the samples we have made, we selected top hBN, which is smaller than bottom hBN, in the heterostructures so that the movement of the top 2D layers can be seen more clearly. However, for purposes of device fabrication, the top hBN is not necessarily always smaller than the bottom hBN. Even if top hBN is larger than the bottom hBN with some small regions touching the silicon substrate, according to our observations, the movement of the top 2D layers is not hindered.

In conclusion, we introduce an in situ manipulation of van der Waals heterostructures mediated by patterning a polymer resist patch onto target flakes, which can precisely and dynamically control the rotation and positioning of 2D materials by a simple gel stamp manipulator. Using this technique, we realized a perfect alignment of graphene with respect to hBN layers with a much higher success rate compared with conventional optical alignment of crystal edges during micromechanical transfer. Our technique can be easily generalized to other 2D material systems (as shown in fig. S6) and allows for reversible manipulation in any 2D systems away from commensurate regime (where it becomes irreversible). We believe our technique has the potential for in situ twistronics using micromanipulators or microelectromechanical systems inside the cryogenic measurement systems, thus opening up a new strategy in device engineering and finding its applications in research of 2D quasi-crystals (*43*, *44*), magic-angle flat bands (*11*–*14*), devices with AB/BA domain walls (*45*), and other topologically nontrivial systems.

## MATERIALS AND METHODS

### Van der Waals assembly

All heterostructures were assembled using standard dry-transfer technique (*46*) using a PMMA coated on a PDMS stamp, and SiO_2_ (290 nm)/Si as a substrate. The devices made from the monolayer and bilayer graphene regions are in a Hall bar geometry, with electrical contacts made by Cr/Au (3 nm/80 nm). The graphene flakes were obtained by mechanical exfoliation of bulk graphite (NGS naturgraphit; Graphenium Flakes, 25 to 30 mm).

### Raman characterization

The Raman spectra were acquired by the Renishaw Raman System with 1800 lines/mm grating, using linearly polarized laser radiation at the wavelength of 532 nm. The laser power was kept below 5 mW. The Raman spatial maps were taken with a step size of 0.5 μm. To extract the peak width of the 2D band, we fit the spectrum at each pixel in the spatial mapping to a single Lorentzian function.

### Transport measurements

Transport measurements were carried out in a four-terminal geometry with a low-frequency ac excitation of 100 nA using standard lock-in technique at the base temperature of 0.3 K (BZ oscillations were measured at 70 K). The devices were gated to control a charge carrier density (*n*) and a displacement field (*D*) by applying bias voltages to the metal top gate (*V*_t_) and the doped silicon substrate (*V*_b_). The charge carrier density is determined by *n* = (*D*_b_ − *D*_t_)/*e*, the vertical displacement field *D* is set by *D* = (*D*_b_ + *D*_t_)/2. Here, *D*_b_ = ε_𝑏_(*V*_b_ − *V*_b_^0^)/*d*_b_, and *D*_t_ = −ε_t_(*V*_t_ − *V*_t_^0^)/*d*_t_, where ε_t,b_ and *d*_t,b_ are the dielectric constants and thicknesses of the top and bottom dielectric layers, respectively, and *V*_b_^0^ and *V*_t_^0^ are the effective offset voltages caused by environment-induced doping.

### Calculation of moiré wavelength, twist angle, and super-moiré wavelength

The moiré wavelength λ is calculated using the geometric relation $n_{s}=4/\frac{\sqrt{3}}{2}\lambda^{2}$, where *n*_s_ is the charge density at full filling of the moiré miniband (at the density of four electrons per superlattice unit cell). The relation between twist angle θ and moiré superlattice wavelength λ in the hBN/graphene/hBN heterostructure is given by (*47*)

$$\begin{array}{c} \lambda =\frac{(1+\delta )a}{\sqrt{2(1+\delta )(1-\text{cos}\theta )+\delta^{2}}}\\ \text{tan}\;\varphi =\frac{\text{sin}\theta }{(1+\delta )-\text{cos}\theta } \end{array}$$

where *a* is the graphene lattice constant; δ is the lattice mismatch between hBN and graphene, which is 1.65% [we used the value calculated by (*34*, *39*)]; and φ is the relative orientation of the two moiré patterns with respect to the graphene lattice (φ_1_ and φ_2_, respectively).

Using the method described previously (*38*), for super-moiré wavelength Λ, in analogy to the equation above, the relation between Λ and the twist angle Θ between the constituent moiré patterns (the wavelengths being λ_1_ and λ_2_; λ_1_ ≥ λ_2_) is given by

$$\Lambda =\frac{(1+\Delta )\lambda_{2}}{\sqrt{2(1+\Delta )(1-\text{cos}\Theta )+\Delta^{2}}}$$

where Δ is the mismatch between the constituent moiré patterns, given by

$$\Delta =\frac{\lambda_{1}-\lambda_{2}}{\lambda_{2}}$$

and the twist angle Θ is given by

$$\Theta =(|\varphi_{1}-\varphi_{2}|-30{}^{\circ})\text{mod}(60{}^{\circ})-30{}^{\circ}$$

The modular division used here to find Θ reproduces the output of the piecewise conditional reported in (*38*).

## Supplementary Material

http://advances.sciencemag.org/cgi/content/full/6/49/eabd3655/DC1

Movie S1

Movie S2

Adobe PDF - abd3655_SM.pdf

In situ manipulation of van der Waals heterostructures for twistronics

## SUPPLEMENTARY MATERIALS

Supplementary material for this article is available at http://advances.sciencemag.org/cgi/content/full/6/49/eabd3655/DC1
