# Supplementary material for: In situ manipulation of van der Waals heterostructures for twistronics
Source: Sci Adv. 2020 Dec 4;6(49):eabd3655. doi: 10.1126/sciadv.abd3655 (PMC7717928; doi:10.1126/sciadv.abd3655)
Supplement: http://advances.sciencemag.org/cgi/content/full/6/49/eabd3655/DC1 [file supp_6_49_eabd3655__index.html]

Science Advances | Science AdvancesAAASSearchScience AdvancesMenu

## Supplementary Materials

# In situ manipulation of van der Waals heterostructures for twistronics

Yaping Yang, Jidong Li, Jun Yin, Shuigang Xu, Ciaran Mullan, Takashi Taniguchi, Kenji Watanabe, Andre K. Geim, Konstantin S. Novoselov, Artem Mishchenko

Download Supplement

**This PDF file includes:**

- Sections S1 to S4
- Figs. S1 to S13
- Table S1
- Legends for movies S1 and S2
- References

**Other Supplementary Material for this manuscript includes the following:**

- Movie S1
- Movie S2

**Files in this Data Supplement:**

- Adobe PDF - abd3655\_SM.pdf
