# Supplementary material for: In situ manipulation of van der Waals heterostructures for twistronics
Source: Sci Adv. 2020 Dec 4;6(49):eabd3655. doi: 10.1126/sciadv.abd3655 (PMC7717928; doi:10.1126/sciadv.abd3655)
Supplement: http://advances.sciencemag.org/cgi/content/full/6/49/eabd3655/DC1 [file supp_6_49_eabd3655__1.pdf]

Science Advances
AAAS

advances.sciencemag.org/cgi/content/full/6/49/eabd3655/DC1

Supplementary Materials for

In situ manipulation of van der Waals heterostructures for twistronics

Yaping Yang*, Jidong Li, Jun Yin, Shuigang Xu, Ciaran Mullan, Takashi Taniguchi, Kenji Watanabe, Andre K. Geim, Konstantin S. Novoselov, Artem Mishchenko*

*Corresponding author. Email: ypyang0916@gmail.com (Y.Y.); artem.mishchenko@gmail.com (A.M.)

Published 4 December 2020, *Sci. Adv.* **6**, eabd3655 (2020)
DOI: 10.1126/sciadv.abd3655

The PDF file includes:

Sections S1 to S4
Figs. S1 to S13
Table S1
Legends for movies S1 and S2
References

Other Supplementary Material for this manuscript includes the following:

(available at advances.sciencemag.org/cgi/content/full/6/49/eabd3655/DC1)

Movies S1 and S2

Supplementary information

1. Identification of the initial contact between polymer gel and epitaxial polymer

In our manipulation technique, the critical step to precisely control the movement of 2D flakes is the identification of the initial contact between the polymer gel (PDMS) and the epitaxial polymer patch (PMMA). The signature of the contact is a color change in the PMMA patch and an appearance of a sharpened edge of the contact area between the PDMS gel and PMMA patch, which is easily distinguished under optical microscope, Fig. S1.

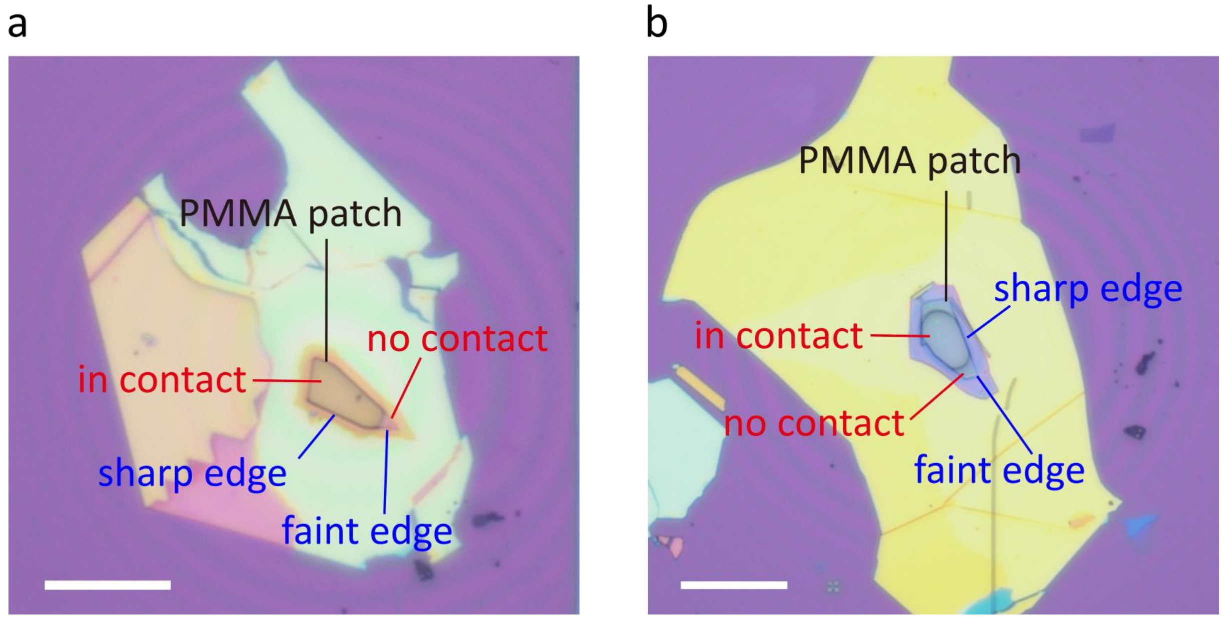

Fig. S1. Micrographs showing the moment when polymer gel (PDMS) is brought into contact with epitaxial polymer (PMMA). (a) PDMS gel is in contact with most of the PMMA patch, only in a small region on the right corner the contact is absent. The region in contact shows a different color from the no-contact region. (b) PDMS gel touches the center of the PMMA patch, with the in-contact area limited to the PMMA patch. In both images, the edges of the in-contact regions are thicker than the edges of no-contact regions. The scale bars are 50 µm.

2. The motion of incommensurately stacked 2D materials

In our manipulation technique, the motion of 2D materials in the van der Waals heterostructure depends on the balance between external driving force and the adhesion between PMMA patch and the top 2D layer, as well as the kinetic friction originating from the van der Waals force between the adjacent 2D layers. The kinetic friction between 2D layers is dramatically reduced in the incommensurate state due to the cancellation of lateral corrugation forces between two crystals in the sliding direction, the so called superlubricity where the 2D flakes can move smoothly. Inversely, in commensurate state, the two adjacent 2D layers are locked together due to pinning of the boundaries that separate local regions of the commensurate phase (*25, 29, 30*), thus the friction is significantly higher. Such boundaries, either topological defects or misfit dislocations, are caused by the lattice mismatch and twist angle between adjacent layers.

The sliding of crystalline interfaces is governed by the friction between the layers. The kinetic friction between graphene and hBN shows rotational anisotropy and six-fold symmetry with respect to the rotation

angle between graphene and hBN (*48*). In incommensurate state where kinetic friction is extremely small, it still varies slightly with the rotation angle (*48*). Therefore, the friction forces at graphene/top hBN and graphene/bottom hBN interfaces are comparable but slightly different from each other depending on the twist angles θ_t and θ_b, which could lead to simultaneous rotation of both top hBN and graphene. Most likely, due to the superlubricity, as the top hBN rotates under the control of PMMA patch, graphene remains static until top hBN clicks to it, and then with further rotation, the top hBN together with graphene clicks to bottom hBN. More realistically, the interface conditions vary among the whole stack. There could be polymer residues or other contaminants adsorbed on the surface of bottom hBN, causing difference in frictions at the two interfaces, thereby graphene can rotate with top hBN simultaneously.

For graphene/hBN and hBN/hBN interfaces, the commensurate state occurs at a small twist angle between the layers, since these two interfaces already satisfy the condition of a small lattice mismatch (1.65% (*34, 39*) for graphene/hBN interface and 0% for hBN/hBN interface). At commensurate state, the locking of the graphene/hBN and hBN/hBN interfaces limits further motion of the flakes (*33*), thereby when the external driving force is large enough, the PMMA patch/hBN/graphene/hBN stack will break at the weaker coupled PMMA patch/hBN interface, in agreement with the delamination of PMMA patch when graphene is aligned to both hBN layers after rotation (Fig. 2e in the main text and Fig. S4c). By slightly etching the top hBN and then depositing the polymer patch, the adhesion between PMMA and the top hBN can be dramaticaly improved and PMMA will not delaminate from the top hBN. Then, if one imposes a larger external driving force in order to further rotate or move the crystals after locking, the 2D crystals will be destroyed, as shown in Fig. S2, where we used hBN/graphite flake/hBN stack as an example. Rotation of the top two layers of hBN/graphene/hBN heterostructure with different initial settings of θ_t and θ_b should result in the final stack with commensurate or incommensurate states at the two interfaces, as shown in Fig. S3 and Table S1. The scenarios are based on the fact that graphene can rotate with top hBN simultaneously. Here we consider both the 0° and 60° alignment of graphene and hBN layer, since these two types of alignment have distinct symmetry and affect electronic structure of graphene differently (*34, 41*).

We fabricated hBN/graphene/hBN heterostructure (sample 2) with the manipulation process matching the cases in Fig. S3b (initial alignment) and Fig. S3j (final alignment). To make sure that the crystal orientation of the flakes is known, we first identified hBN fakes which have fractured into two pieces during the mechanical exfoliation procedure (Fig. S4a). Note that for hBN crystals with odd numbers of layers, the top and bottom layers have the same crystal orientation, whereas for hBN crystals with even numbers of layers, the top and bottom layers are of mirror symmetry (Fig. 2c in the main text). Therefore, the common edges of the fractured hBN pieces indicate the same crystal orientation of the atomic layer belonging to the same surface, either top or bottom, of the original crystal. Then we used one of the fractured hBN piece to pick up graphene and flipped over the other fractured piece as the bottom hBN layer, so that the hBN atomic layers adjacent to graphene come from the same crystal surface. See Fig. S5 for the details of this procedure. Next, the initial θ_t and θ_b were set according to Fig. S3b and the following rotation direction led to the final stack with $\theta_t=0^o$, $\theta_b=\theta_{tb}=60^o$ (Fig. S4c), which is the case of Fig. S3j.

We carried out Raman spectroscopy to prove the alignment of graphene and hBN in sample 2, as shown in Fig. S4d and e. The behavior of Raman spectra in monolayer and bilayer graphene regions are similar to those observed in sample 1. Whereas the full width at half maximum of 2D peak ($FWHM_{2D}$) increased from 17 cm^{-1} to 48.5 cm^{-1} after rotation, smaller than that in the perfect alignment case, which means that the twist angles θ_t and θ_b are larger than those in sample 1. Figure S4e shows the distribution of $FWHM_{2D}$ among the graphene flake after rotation, indicating a spatially uniform twist angle in both monolayer and bilayer regions. The AFM topography (Fig. S4f) here shows that the rotation process did not damage or crease the graphene layer.

The assembly process of sample 2 is illustrated in Fig. S5. The details of the process are as follows. **Step 1**, we used PMMA coated PDMS block mounted on a glass slide to pick-up one of the fractured hBN pieces as the top hBN (hBN piece 1). **Step 2**, we used polypropylene carbonate (PPC) coated PDMS block mounted on another glass slide to pick-up the other fractured hBN piece (hBN piece 2), which serves as the bottom hBN. **Step 3**, the target graphene flake is picked up by PDMS/PMMA/hBN piece 1. Now the graphene flake is in contact with the bottom surface of hBN piece 1. **Step 4**, the PDMS/PPC block is flipped over so that the bottom surface of hBN piece 2 faces upwards, as indicated by the red line in the figure. Then the PDMS/PMMA/ hBN piece 2 is rotated in order to align the shared crystal edges of hBN piece 1 and 2 under microscope. **Step 5**, hBN piece 2 is picked up by PMMA/hBN piece 1 /graphene at temperature above 75^0. When temperature is high enough, the adhesion between PPC and hBN is smaller than the adhesion between PMMA and hBN. Thereby the bottom hBN (hBN piece 2) can be easily picked up by PMMA/hBN piece 1 /graphene. **Step 6**, the final stack hBN piece 1/graphene/ hBN piece 2 is released onto a new silicon wafer by dissolving away PMMA using acetone. In the final stack, graphene flake is in contact with both the bottom surfaces of hBN piece 1 and hBN piece 2. Since the bottom surfaces of hBN piece 1 and hBN piece 2 are originally from the same atomic layer, we can guarantee the settings of θ_t and θ_b if the alignment of the shared edges is based on the same surface of the fractured hBN pieces.

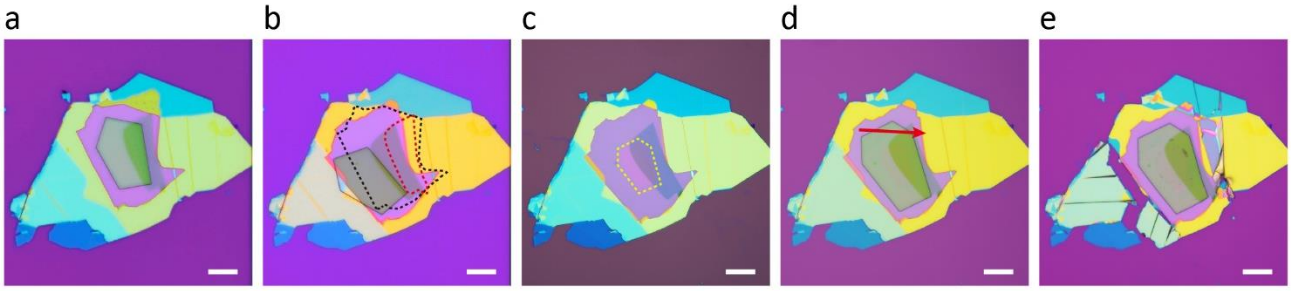

Fig. S2. Displacement of hBN/graphite/hBN stack after commensurate locking. (a) hBN/graphite/hBN stack deposited with PMMA patch. (b) Top hBN and graphite rotated with PMMA patch, and then PMMA patch delaminated from top hBN after the 2D layers lock with each other. The black and red dashed polygons indicate the original position of top hBN and graphite, respectively. (c) Slightly etched top hBN. The yellow dashed polygon indicates the etched region, which shows color difference from non-etched region. (d) A new PMMA patch deposited onto the top hBN, covering the etched region. The red arrow implies the displacement direction. (e) Bottom hBN was broken after the displacement, without delamination of PMMA patch. The scale bars are 20 μm.

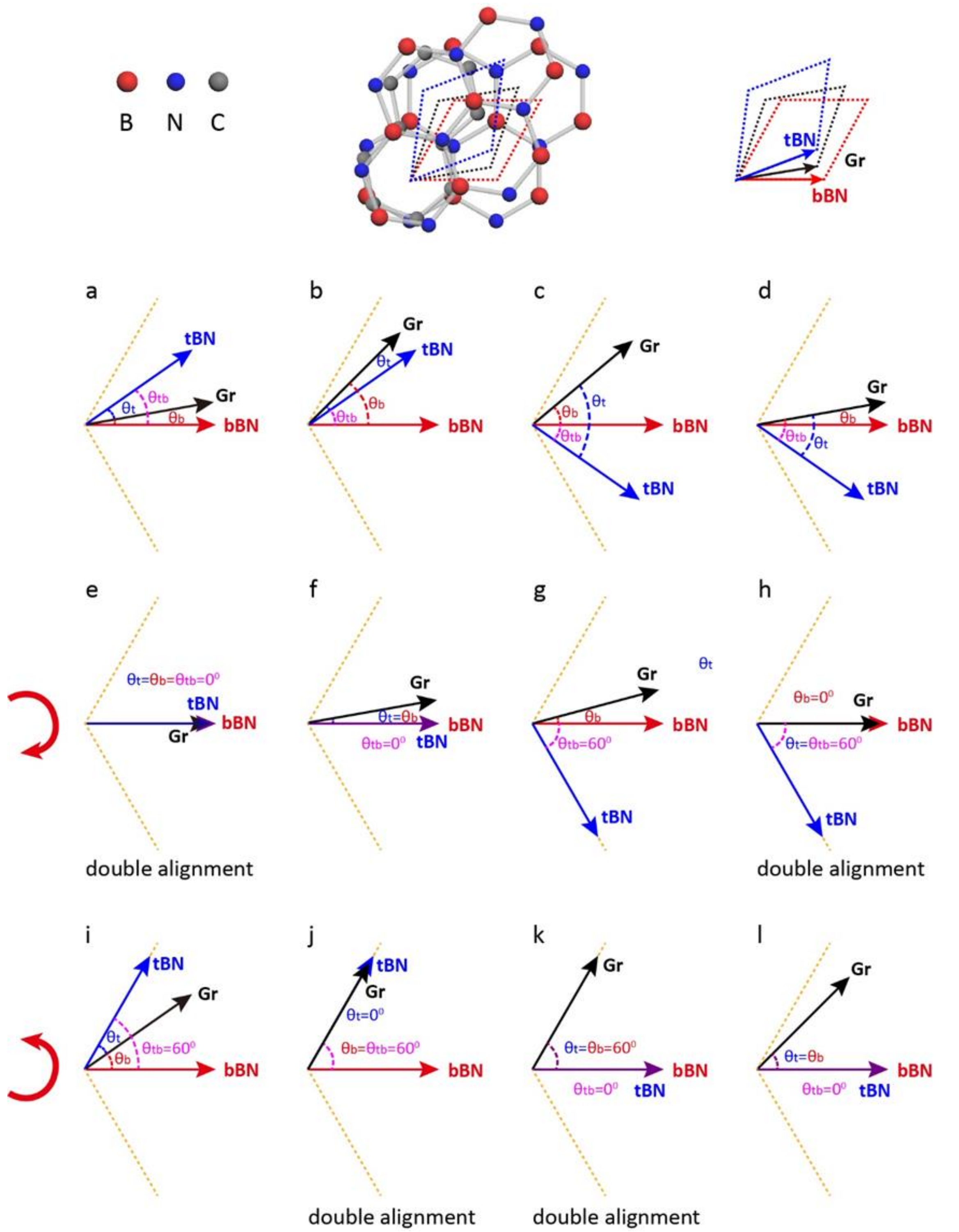

Fig. S3. The resulting alignment of hBN/graphene/hBN stack under different rotation directions. (a) to (d) hBN/graphene/hBN stack with different initial settings of θ_t and θ_b. (e) to (h) The final stacks corresponding to (a) to (d) with different types of alignment after clockwise rotating. (i) to (l) The final stacks corresponding to (a) to (d) with different types of alignment after anti-clockwise rotating. The red, black and blue arrows indicate the crystal orientation of bottom hBN, graphene and top hBN, respectively. The red arc arrows on the left show the rotation direction. During the rotation process, the bottom hBN layer remains at original position and only the top hBN and graphene rotate under the control of polymer gel manipulator. Orange dashed lines mark ±60° with respect to red arrows (bottom hBN), the rotations will stack at these lines as top hBN goes into AA' stacking with bottom hBN.

Table S1. Resulting stack with different initial settings of θ_t and θ_b as shown in Fig. S3.

Panels in Fig. S3	Initial setting of twist angles (°)	Resulting stack — Rotation direction: clockwise	Resulting stack — Rotation direction: anticlockwise
a	$0<\theta_t,\ \theta_b<\theta_{tb}<60^o$	$\theta_t=0^o,\ \theta_b=0^o,\ \theta_{tb}=0^o$ double alignment	$\theta_t\neq0^o, \theta_b\neq0^o,$ $\theta_{tb}=60^o$, misalignment
b	$0<\theta_t,\ \theta_{tb}<\theta_b<60^o$	$\theta_t=\theta_b\neq0^o,\ \theta_{tb}=60^o$, misalignment	$\theta_t=0^o,\ \theta_b=\theta_{tb}=60^o$, double alignment
c	$-60^o<\theta_{tb}<0<\theta_b<60^o,\ \lvert60^o-\theta_{tb}\rvert<\theta_b$	$\theta_t=\theta_b\neq0^o,\ \theta_{tb}=60^o$, misalignment	$\theta_t=\theta_b=60^o,\ \theta_{tb}=0^o$, double alignment
d	$-60^o<\theta_{tb}<0<\theta_b<60^o,\ \lvert60^o-\theta_{tb}\rvert>\theta_b$	$\theta_b=0^o,\ \theta_t=\theta_{tb}=60^o$, double alignment	$\theta_t=\theta_b\neq0^o,\ \theta_{tb}=0^o$, misalignment

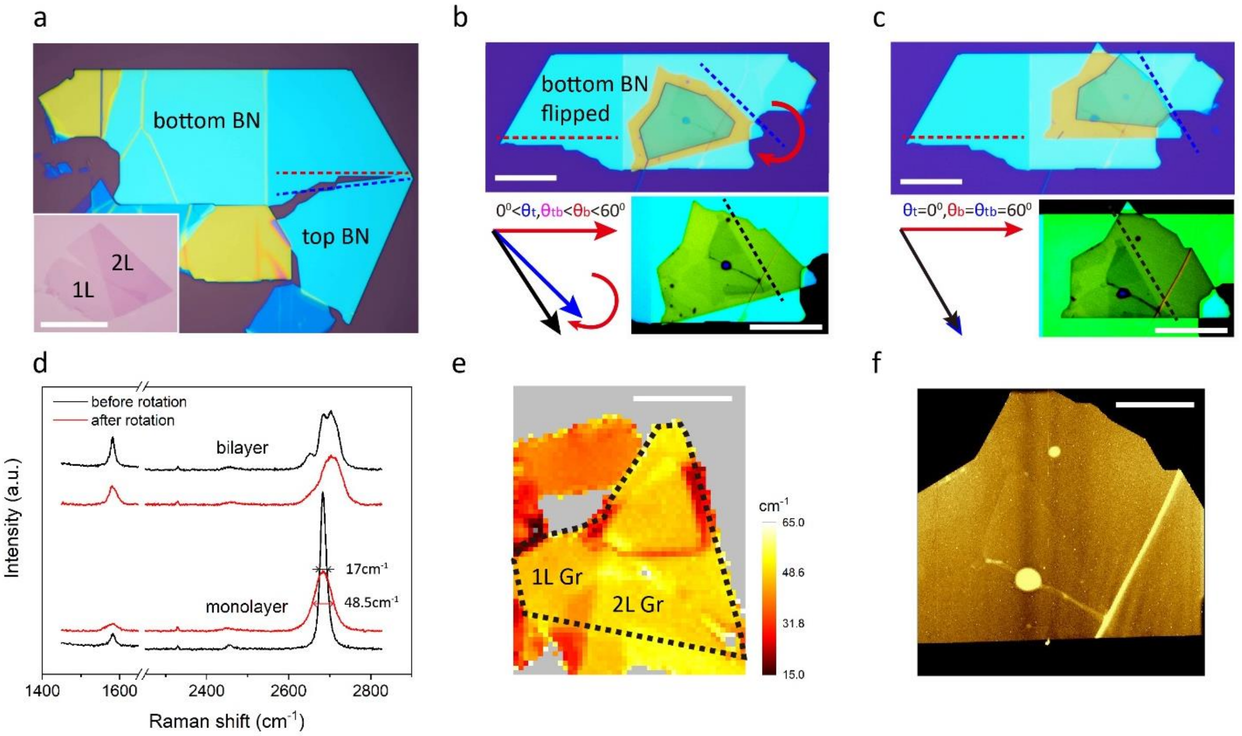

Fig. S4. Encapsulated graphene aligned to both top and bottom hBN, with $\theta_t=0^o$, $\theta_b=\theta_{tb}=60^o$. (a) Optical image of an hBN flake with a crack after mechanical exfoliation. The bottom inset shows the graphene flake containing monolayer and bilayer region. (b) Bottom hBN was flipped over before the release of top hBN/graphene stack. The final heterostructure was intentionally designed with the relative crystal orientations $0<\theta_t$, $\theta_{tb}<\theta_b<60^o$, as shown in left bottom inset, which matches the case in Fig. S3b. The red arc arrow implies the rotation direction. (c) The heterostructure after rotation, with $\theta_t=0^o$, $\theta_b=\theta_{tb}=60^o$ as shown in left bottom inset, which matches the

case in Fig. S3j. The PMMA patch delaminated from top hBN after all the crystals were aligned to each other. The right bottom insets of (b) and (c) increase the contrast to show the position of graphene. The red, blue and black dashed lines in (a-c) indicate the crystal orientation of bottom hBN, top hBN and graphene respectively. (d) Raman spectra of graphene at the monolayer and bilayer region before and after rotation, respectively. (e) 2D band width Raman map of the stack after rotation. The dashed line shows the region where graphene is covered by top hBN. (f) AFM topography of the stack after rotation. The scale bars in (a) to (c) are 20 µm. The scale bars in (e) and (f) are 10 µm.

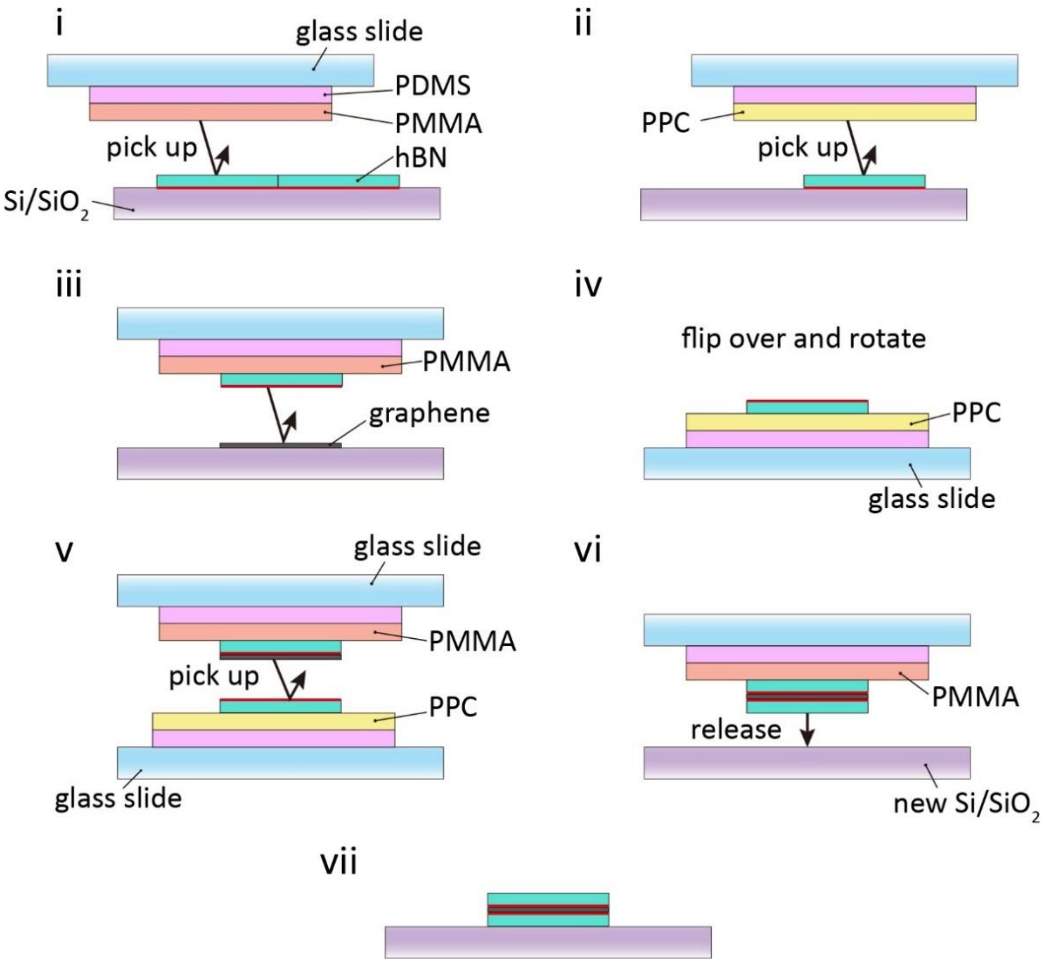

Fig. S5. Schematic process flow for graphene encapsulated by the same hBN crystal. In the final stack, the hBN atomic layers adjacent to graphene belong to the same surface of the original crystal, as highlighted by the red lines.

For other interfaces where the adjacent layers have larger lattice mismatch, such as MoS_2/hBN interface, a small or even zero twist angle will not result in the commensurate state. The kinetic friction between the 2D layers always remains at an extremely low value, thereby the 2D flake can move and rotate smoothly even when passing through the point where twist angle is zero without the delamination of PMMA patch, as shown in Fig. S6.

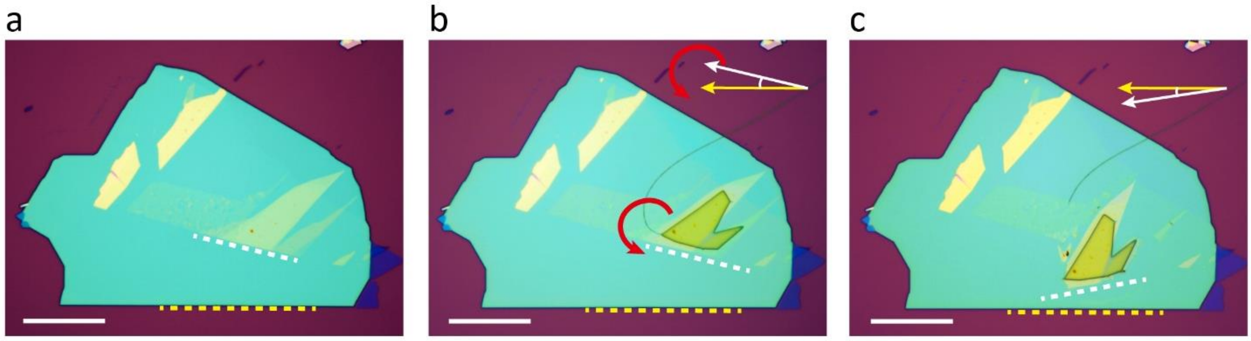

Fig. S6. Rotation of MoS_2 on top of hBN. (a) Optical image of MoS_2/hBN stack before rotation. The white and yellow dashed lines indicate the crystal orientations of MoS_2 and hBN crystal, respectively. (b) PMMA patch patterned onto MoS_2 flake. The white and yellow arrows show the twist angle between MoS_2 and hBN crystal. The red arc arrow implies the rotation direction. (c) Rotation of MoS_2 flake with the twist angle passing through 0°, without PMMA being delaminated from MoS_2 flake, confirming that there is no commensurate state at a small twist angle of MoS_2 and hBN crystal. The scale bars are 20 μm.

3. Raman spectra of graphene in the monolayer and bilayer regions before and after rotation

Raman spectra of graphene are strongly modified when graphene is subjected to double moiré superlattices (Fig. 2g). In our sample, for monolayer graphene region, G peak changes its shape from a narrow peak centered at 1582 cm^{-1} with $FWHM_G$ ≈14.5 cm^{-1} to a broadened asymmetric peak after rotation. The presence of a low energy shoulder at ≈1558 cm^{-1} of G peak after rotation is attributed to a TO phonon, similar to what was observed in graphene aligned to only one hBN crystal (*35*). For 2D peak, the broadening effect is strongly enhanced in double-moiré superlattice compared to a simple aligned graphene/hBN heterostructure: ≈20 cm^{-1} increase in FWHM for graphene in a single moiré superlattice (*27, 35*) and ≈40 cm^{-1} increase in FWHM for our doubly-aligned sample, indicating that the double moiré superlattices induce a much stronger periodic inhomogeneity originated from charge accumulation, strain, etc.

For bilayer graphene region, G peak splits into two components in the presence of double moiré superlattices. For 2D peak, the four components broadened and their position differences reduced, which is similar to what was reported in bilayer graphene aligned to only one hBN crystal (*49*).

We found an overall downshift of around 2cm^{-1} for both G peak and 2D peak among the whole flake after rotation (Fig. S7 a-d), which contradicts the results of previous studies (*27, 34*). Similar behavior is found in Sample 2 (Fig. S8). Near the corner of the folded region (as indicated by the arrows), before rotation, the peak positions of G and 2D peaks are slightly lower than those of the nearby region. We attribute this phonon softening to the strain caused by folding (*50*). Whereas after rotation, peak position near the corner of the folded region shows an upshift behavior, and is higher than that of the nearby region. Note that during the rotation, the folded graphene rotated as well, therefore we expect an enhanced strain effect near this region. However the abnormal behavior of the peak position implies a mechanism beyond strain effect.

To further look into the line shape of G peak after rotation, we fit G peak with two Lorentzian peaks for

both bilayer and monolayer graphene regions and plot the maps of the peak position difference and intensity ratio of the two components, as shown in Fig. S7e and f. The maps show that the G peak splitting is homogeneous for both bilayer and monolayer graphene regions, which is around 13 cm^{-1} and 22 cm^{-1}, respectively. The intensity ratio of the two components is around 1 for the bilayer region, which means that they have comparable weight, and is around 0.6 for the monolayer region.

The overall downshift and broadening of the peak positions for G and 2D peaks and splitting of G peak remind one of the effects of strain. Under uniaxial strain, the doubly degenerate E_{2g} mode splits into two components, one along the strain direction and the other perpendicular, which leads to the splitting of G peak (*51*). Tensile strain usually gives phonon softening, while compressive strain usually leads to phonon stiffening. In the presence of moiré superlattice, the spatial strain distribution is periodically modulated, and tension and compression of graphene lattice should coexist. The combined effect of the opposite types of strain could explain the observed behavior of the Raman peaks.

Alternatively, the shift of G and 2D peaks could be related to a change in the Kohn anomalies associated with the phonon dispersion at Γ and K. For instance, the aligned hBN layers in the hBN/graphene/hBN heterostructure could have built-in electric fields (*52*) which can dope graphene and thereby shift the Raman peak positions. However, this scenario is unlikely, as previous studies show that the presence of electrostatic doping causes non-adiabatic removal of the Kohn anomaly from the Γ point, leading to the stiffening of G band and 2D band (*53, 54*). Thus, most likely, the main factor that causes the change in Raman spectra is the periodic strain field induced by moiré patterns.

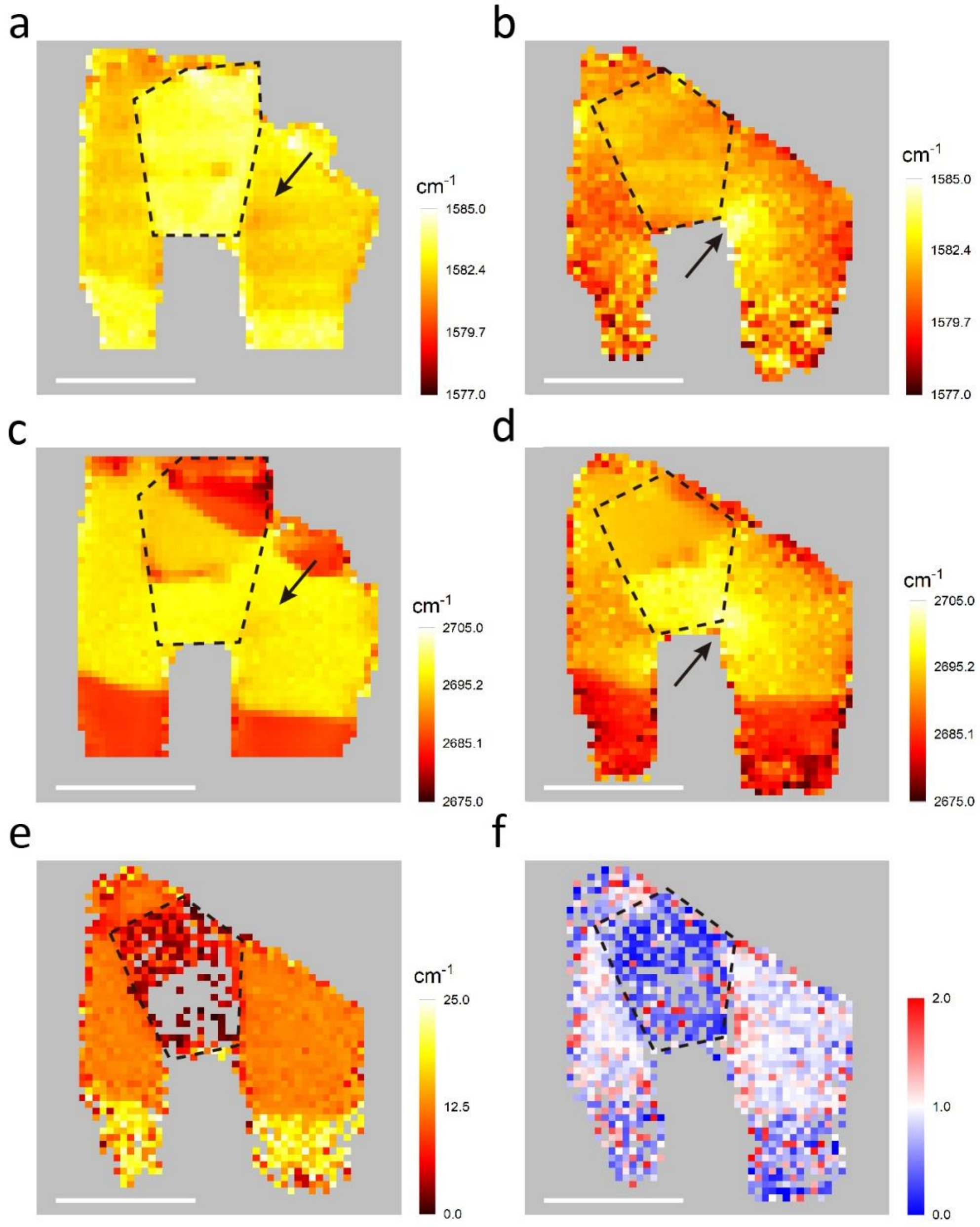

Fig. S7. Raman maps of sample 1 before and after rotation. (a) G peak position map before rotation. (b) G peak position map after rotation. (c) 2D peak position map before rotation. (d) 2D peak position map after rotation. G and 2D peaks of (a) to (d) were fitted by a single Lorentzian. (e) Map of the position difference of the two components of G peak after rotation. (f) Map of the peak intensity ratio of G peak components after rotation. G peak of (e) and (f) was fitted by two Lorentzian peaks. The scale bars are 10 μm. The black dashed lines show the folded region. The black arrows indicate a higher strain level near the folded corner.

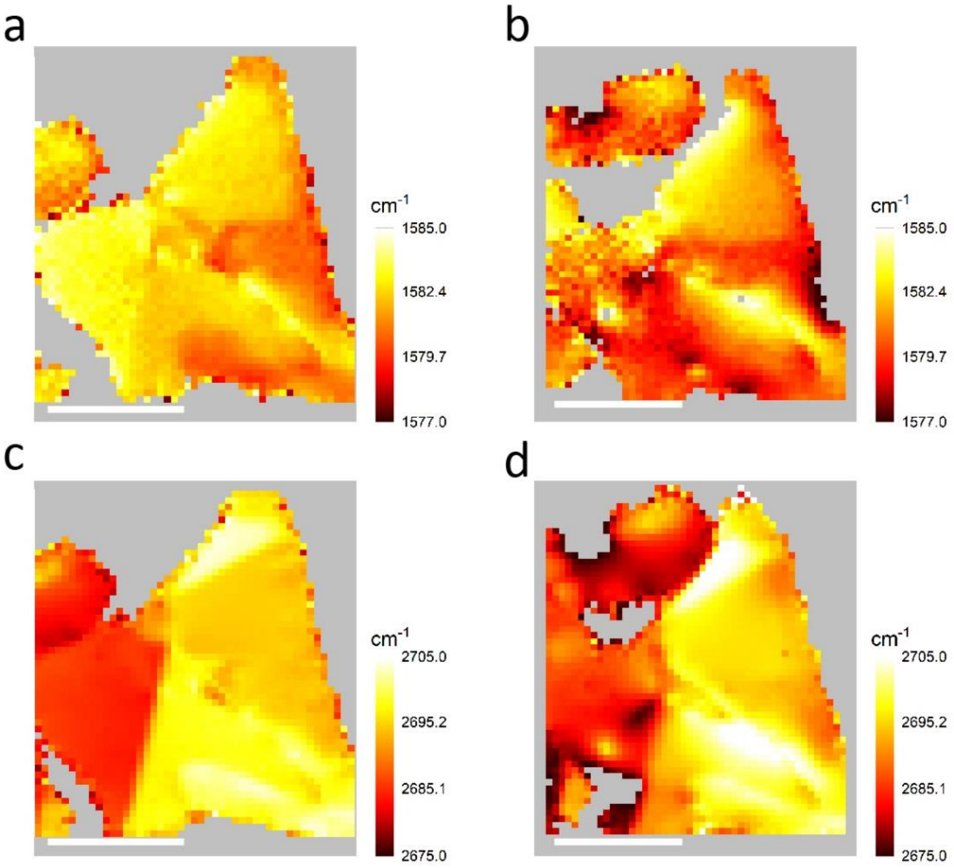

Fig. S8. Raman maps of sample 2 before and after rotation. (a) G peak position map before rotation. (b) G peak position map after rotation. (c) 2D peak position map before rotation. (d) 2D peak position map after rotation. G and 2D peaks were fitted by a single Lorentzian. The scale bars are 10 µm.

4. Transport properties of graphene in double moiré superlattices.

In commensurate magnetic field and periodic moiré potential, the fractal energy spectrum of electron in graphene exhibits a self-similar recursive behavior, where the superlattice minibands show a gapped Dirac-like spectrum at $\phi = \phi_0 p/q$ and express Landau level (LL) characteristics of Dirac fermions (*55*). Therefore we expect to observe quantized Hall conductivity associated with each minigap, similar to what is observed in the usual quantum Hall effect where $\sigma_{xy} = \nu e^2/h$, ν is a filling factor of a particular LL. Accordingly, when σ_{xx} is plotted in a map vs n and B (fan diagram), the gaps between Landau levels (the zeroes in the σ_{xx}) should trace linear trajectories emerging from primary Dirac point (PDP) as well as secondary Dirac points (SDPs) induced by moiré superlattice. For the fans emerging from PDP, the trajectories follows $B = n\phi_0/\nu$. For the fans emerging from SDPs, substituting ϕ with BA and n_0=1/A in the Diophantine equation $\phi/\phi_0 = (n/n_0\text{-}s)/t$ yields $B\text{-}(\text{-}s/t)\phi_0/A = n\phi_0/t$ (which can be viewed as the quantization of minibands at an effective magnetic field of $B_{eff} = B\text{-}(\text{-}s/t)\phi_0/A$ or $B_{eff} = B\text{-}(p/q)\phi_0/A$, where p and q are co-prime integers). Thus we can deduce the corresponding carrier density to SDP from fan diagram.

Based on the Raman spectra of the hBN/graphene/hBN heterostructure, we estimate that the twist angles θ_t and θ_b should be both close to 0°, thereby in the transport properties, we expect to see two sets of SDPs close to each other apart from the PDP. For both bilayer and monolayer graphene devices, in longitudinal resistivity (ρ_{xx}) vs carrier density (n) dependence, we observed broadened (and with a complex structure) satellite peaks located around $n = \pm 2\times 10^{12}$ cm^{-2} (Fig. 3a in the main text and Fig. S11a). At small B = 0.03 T where the Landau quantization is not yet developed, the transversal resistivity ρ_{xy} changes sign in the carrier density regions of these satellite peaks, indicating that they are moiré superlattice induced SDPs. These SDPs are more prominent in the hole side compared to the electron side, which is consistent with previous studies (*5, 6*).

In the Landau fan diagram at high magnetic fields, by tracing the linear trajectories we observed two sets of SDPs induced by the two independent moiré patterns for both bilayer and monolayer device. For bilayer device, the SDPs are at $n_{s1} = \pm 2.15\times 10^{12}$ cm^{-2}, and $n_{s2} = \pm 2.34\times 10^{12}$ cm^{-2} (Fig. 3a, b and d in the main text and Fig. S9a, c and d), corresponding to the moiré wavelengths of λ_{s1} = 14.7 nm and λ_{s2} = 14.0 nm, and the twist angles of 0.24^o and 0.38^o, respectively. For monolayer device, the SDPs are at $n_{s1} = \pm 2.10\times 10^{12}$ cm^{-2}, $n_{s2} = \pm 2.44\times 10^{12}$ cm^{-2} (Fig. S11 and Fig. S12a, c and d), corresponding to the moiré wavelengths of λ_{s1} = 14.8 nm and λ_{s2} = 13.8 nm, and the twist angles of 0.2^o and 0.43^o, respectively. In addition to the Raman map in Fig. 2h in main text, the similar values of the two sets of SDPs obtained from the bilayer and the monolayer devices, confirm that top and bottom twist angles are spatially uniform in the stack.

In the fan diagram $\partial\sigma/\partial B(n,B)$, we observed Brown-Zak (BZ) oscillations originating from the first order magnetic Bloch states (ϕ/ϕ_0=1/q) for the two sets of SDPs in both bilayer and monolayer devices (Fig. S9b, e, f and Fig. S12b, e, f). The first order magnetic Bloch states formed by the two moiré superlattices are also prominent at high temperature (T = 70 K, where the Landau quantization is suppressed), as indicated by the arrows in Figs. S10 and S13. We also observed high order magnetic Bloch states ($\phi/\phi_0 = 3/q$) belonging to the two moiré superlattices, as shown in Fig. S9e, f and Fig. S12e, f.

In principle, the super-moiré pattern generated by the two original moiré patterns should have six possible reciprocal lattice vectors, as described in the previous study (*39*), whereas the method in Ref (*38*) only considers one possible reciprocal lattice vector with the largest super-moiré pattern wavelength (λ_{sm} = 102.3 nm). If we take into account other possible reciprocal lattice vectors, we will get six possible second-order moiré wavelengths in total. For bilayer graphene device, the other five super-moiré wavelengths are 16.3 nm, 12.9 nm, 8.6 nm, 8 nm and 7.2 nm. The corresponding carrier densities n_{sm} of the first Brillouin zone edge are $\pm1.75\times10^{12}$ cm^{-2}, $\pm2.8\times10^{12}$ cm^{-2}, $\pm6.2\times10^{12}$ cm^{-2}, $\pm7.2\times10^{12}$ cm^{-2} and $\pm9\times10^{12}$ cm^{-2}. For monolayer graphene device, the six super-moiré wavelengths are 64.7 nm, 17.8 nm, 12.1 nm, 8.9 nm, 7.8 nm and 7.2 nm. The corresponding carrier densities n_{sm} of the first Brillouin zone edge are $\pm0.11\times10^{12}$ cm^{-2}, $\pm1.46\times10^{12}$ cm^{-2}, $\pm3.14\times10^{12}$ cm^{-2}, $\pm5.8\times10^{12}$ cm^{-2}, $\pm7.5\times10^{12}$ cm^{-2} and $\pm8.9\times10^{12}$ cm^{-2}. We observed satellite peaks in ρ_{xx} near most of these carrier densities n_{sm} (Fig. 3a in the main text and Fig. S11a). Note that near n_{sm} corresponding to these resistivity peaks, most of the low-B ρ_{xy} regions have sign reversal. In addition, similar to bilayer device, in the plot of $\partial\sigma_{xx}/\partial B(n,B)$ of monolayer device, we observe prominent horizontal streaks at B = 1.14 T and 0.57 T, which perfectly matches the magnetic field of the first and second order magnetic Bloch state originating from the super-moiré pattern when $BA = \phi_0$ (where q = 1) and $BA = \phi_0/2$ (where q = 2), respectively. These features allow us to attribute the origin of the observed carrier densities n_{sm} to the super-moiré pattern with different wavelengths.

4.1 Bilayer graphene in double moiré superlattices.

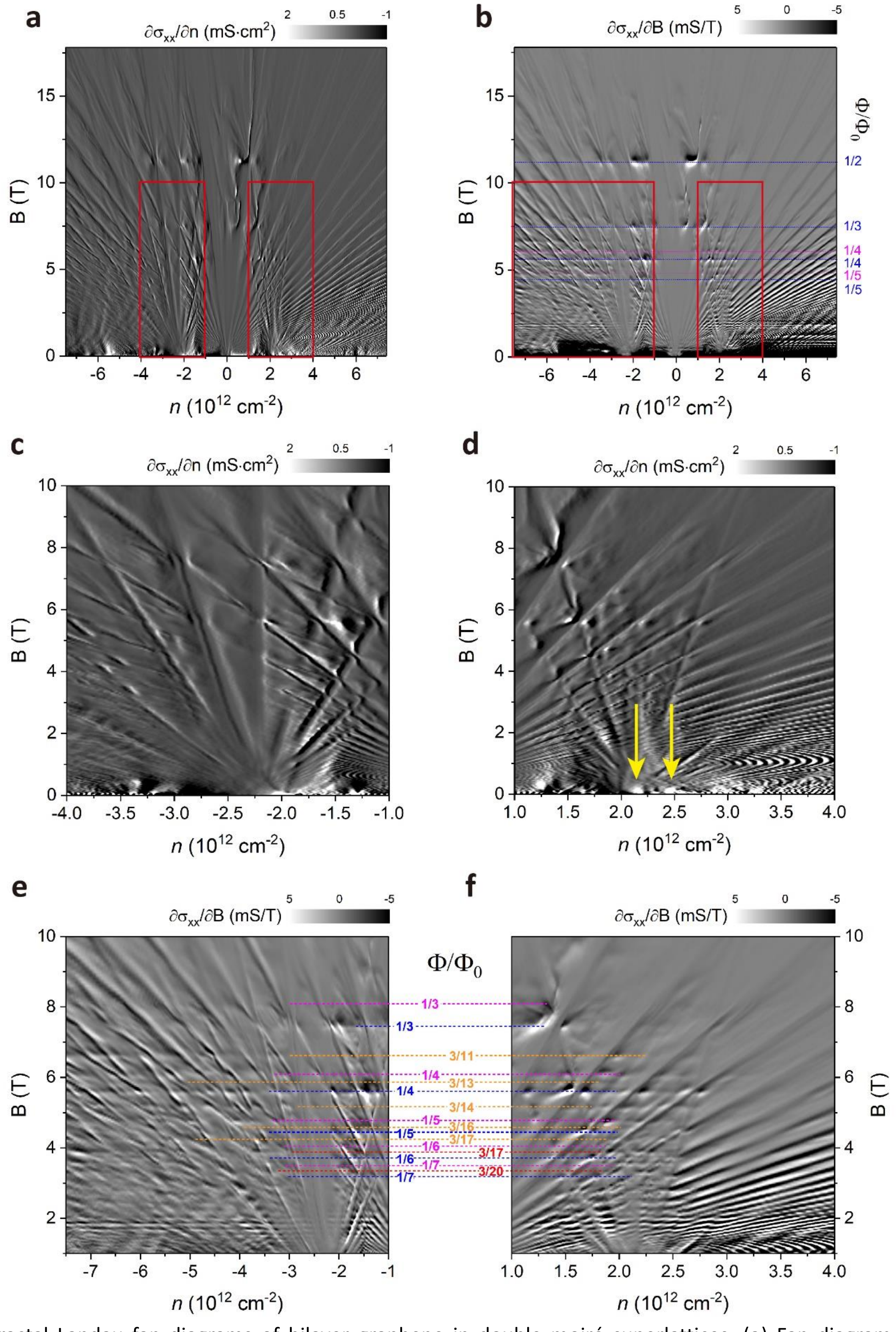

Fig. S9. Fractal Landau fan diagrams of bilayer graphene in double moiré superlattices. (a) Fan diagram $\partial\sigma_{xx}/\partial n(n,B)$ at $T = 0.3$ K. (b) Fan diagram $\partial\sigma_{xx}/\partial B(n,B)$. (c) and (d) are part of (a) for hole and electron doping

(marked in (a) by red rectangles), respectively, near secondary Dirac points (SDPs). Yellow arrows indicate the two sets of SDPs. (e) and (f) are part of (b) for hole and electron doping (marked in (b) by red rectangles), respectively, near SDPs. The blue and red dashed lines show the Brown-Zak oscillations belonging to the SDP at $n_{s1} = \pm 2.14 \times 10^{12}$ cm^{-2}, whereas magenta and orange dashed lines belong to the SDP at $n_{s2} = \pm 2.34 \times 10^{12}$ cm^{-2}. The numbers on the dashed lines show the values of p/q for oscillations at $\phi = (p/q)\phi_0$.

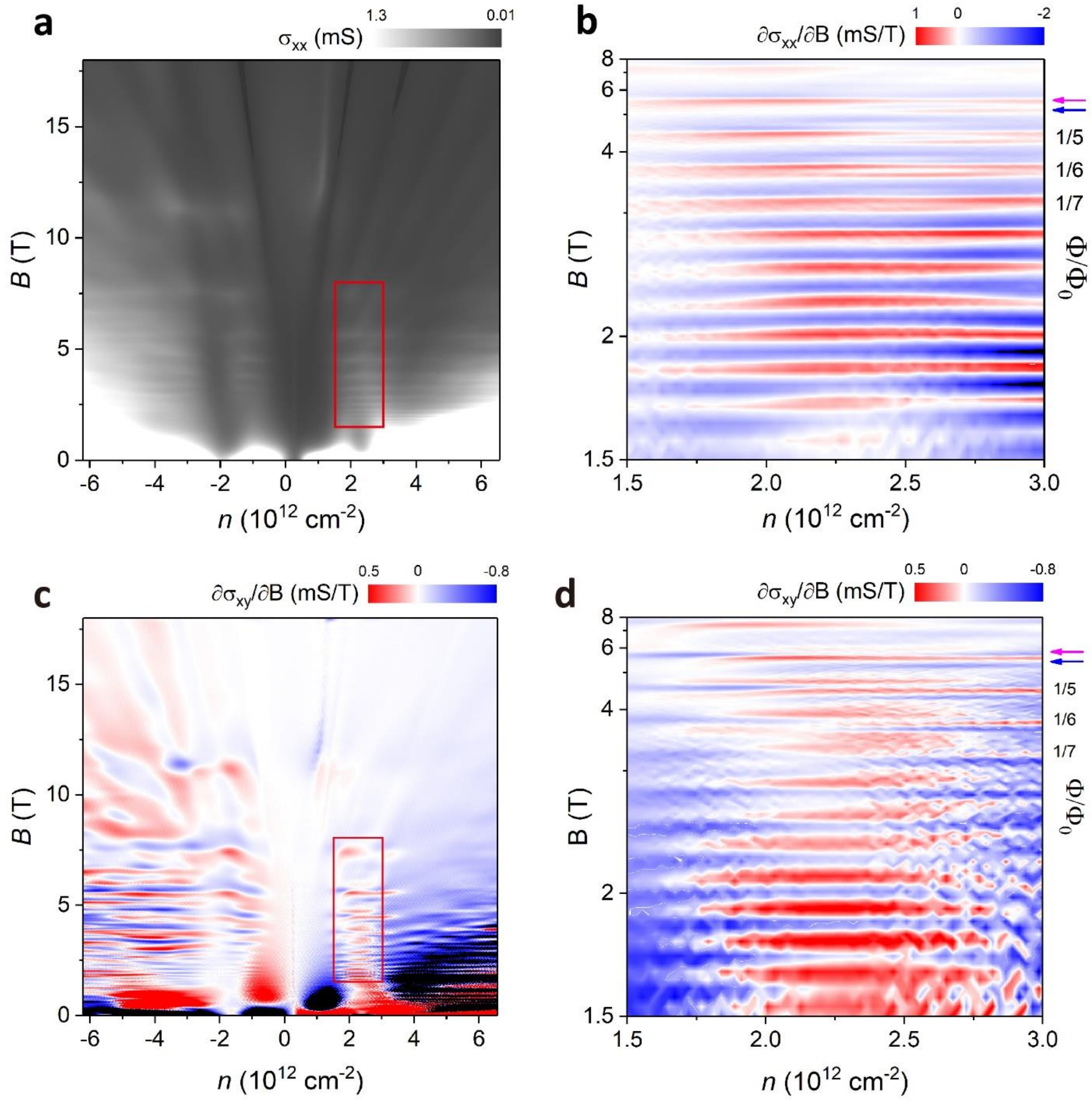

Fig. S10. Brown-Zak oscillations in double moiré bilayer graphene at T = 70 K. (a) Longitudinal conductivity σ_{xx} as a function of n and B, $\sigma_{xx}(n,B)$. (b) $\partial\sigma_{xx}/\partial B(n,B)$ for part of (a) near the SDPs for electron doping (marked in (a) by a red rectangle). (c) $\partial\sigma_{xy}/\partial B(n,B)$. (d) Region of (c) near the SDPs for electron doping (marked in (c) by a red rectangle). The blue and magenta arrows show the Brown-Zak oscillations belonging to the SDPs at n_{s1} = $\pm 2.14 \times 10^{12}$ cm^{-2}, and $n_{s2} = \pm 2.34 \times 10^{12}$ cm^{-2}, respectively. The numbers on the right in (b) and (d) indicate ϕ = $(1/q)\,\phi_0$ with q = 5 to 7. At low magnetic fields, the resolution in B is not sufficient to distinguish the two sets of oscillations.

4.2 Monolayer graphene in double moiré superlattices.

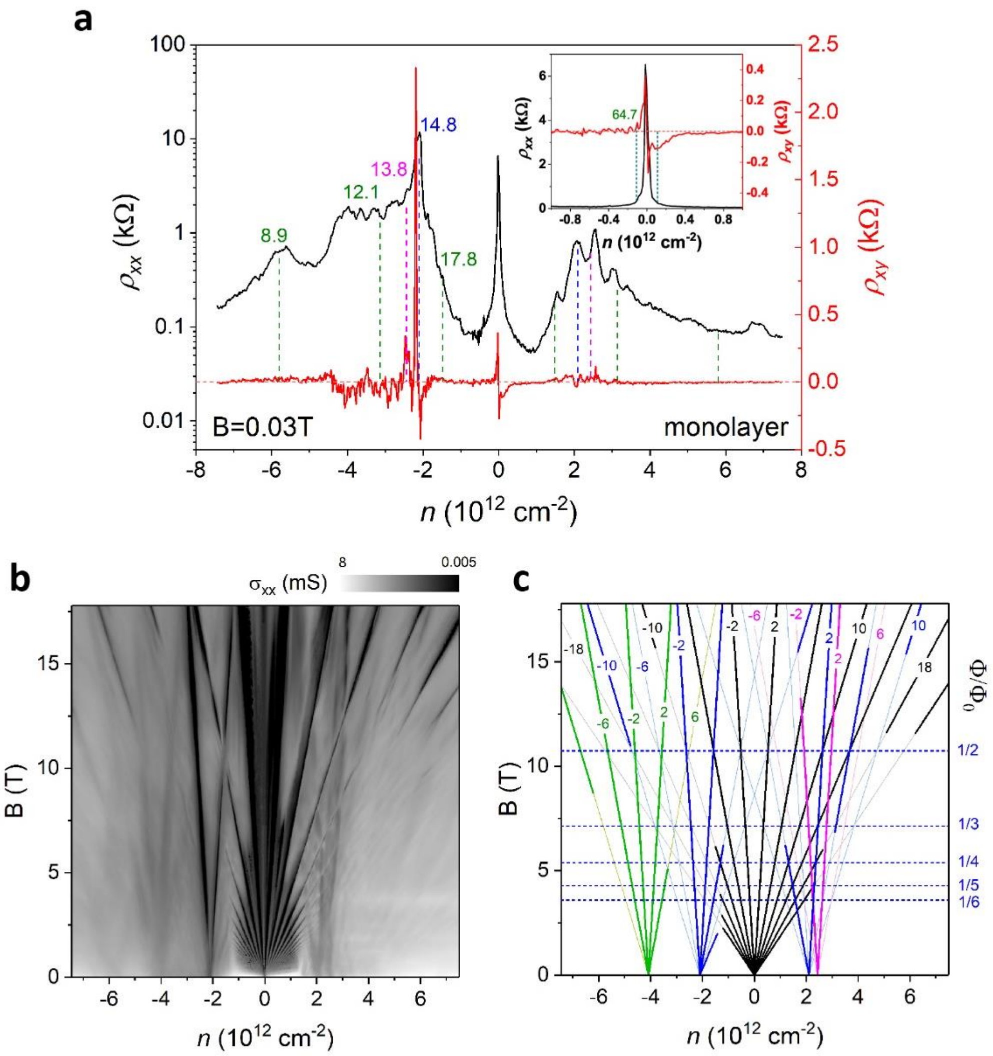

Fig. S11. Electronic transport and Landau quantization of monolayer graphene in double moiré superlattices. (a) Longitudinal resistivity ρ_{xx} and transverse resistivity ρ_{xy} of the monolayer region as a function of charge carrier density n after rotation. T = 0.3 K, B = 0.03 T. The dashed lines and numbers show the satellite peaks and the corresponding moiré wavelengths λ (see Methods). The blue and magenta dashed lines mark the SDPs at n_{s1} and n_{s2} corresponding to moiré patterns formed at both sides of graphene, respectively. The green dashed lines mark the n_{sm} required to reach the first Brillouin zone edge of super-moiré pattern with different wavelengths. The right inset is the zoomed-in figure of (a). (b) Fan diagram $\sigma_{xx}(n,B)$ measured at T = 0.3 K. (c) Simplified Wannier diagram labelling the quantum hall effect states identified in (b). Black lines show quantum oscillations with dominant sequence of Landau level filling factors ν = ±2, ±6, ±10, ... emerging from the PDP. Blue lines show quantum oscillations of t = ±2, ±6, ±10, emerging from SDP n_{s1}. Magenta lines show quantum oscillations of t = ±2 emerging from SDP n_{s2}. Green lines are another set of gap trajectories (s = 2) from the same moiré superlattice as SDP n_{s1} according to the Diophantine equation, with quantum oscillations of t = ±2, ±6. The blue horizontal dashed lines and numbers on the right show the most prominent BZ oscillations belonging to the SDP ns_1, with different values of p/q for ϕ = (p/q) ϕ_0.

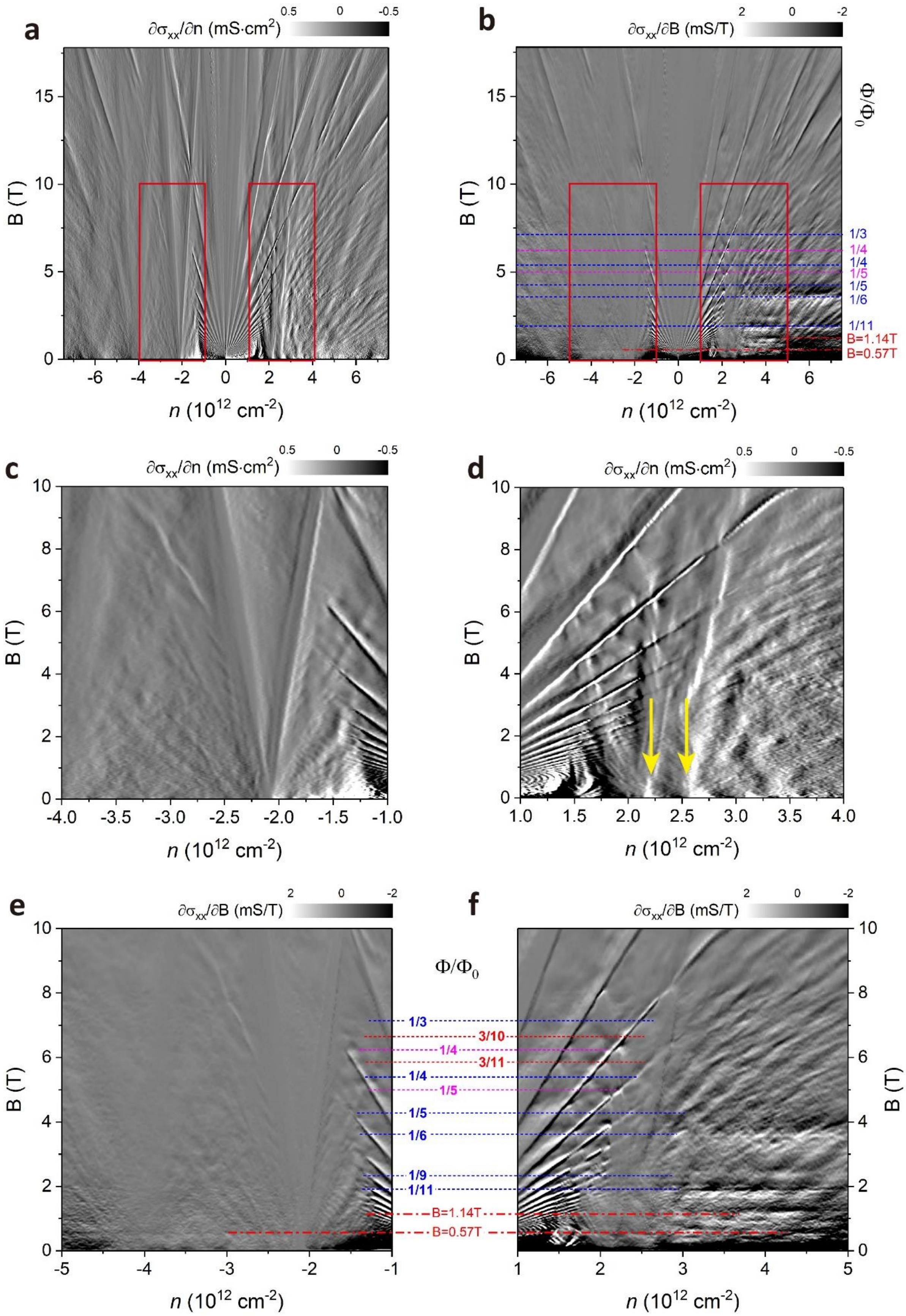

Fig. S12. Fractal Landau fan diagrams of monolayer graphene in double moiré superlattices. (a) Fan diagram $\partial\sigma_{xx}/\partial n(n,B)$ at T = 0.3 K. (b) Fan diagram $\partial\sigma_{xx}/\partial B(n,B)$. (c) and (d) are part of (a) for hole and electron doping

near SDPs (marked in (a) by red rectangles), respectively. Yellow arrows indicate the two sets of SDPs. (e) and (f) are part of (b) for hole and electron doping (marked in (b) by red rectangles), respectively, near SDPs. The blue and red dashed lines show the BZ oscillations belonging to the SDP at $n_{s1} = \pm 2.10\times10^{12}$ cm^{-2}. The magenta dashed lines show the BZ oscillations belonging to the SDP $n_{s2} = \pm 2.44\times10^{12}$ cm^{-2}. The numbers on the dashed lines show the values of p/q for oscillations at $\phi = (p/q)\phi_0$. The BZ feature at B = 1.14 T and 0.57 T originates from the first and second order magnetic Bloch state of the super-moiré pattern with the largest $\lambda_{sm} \approx$ 64.7 nm at $\phi = \phi_0$ and $\phi = \phi_0/2$, respectively.

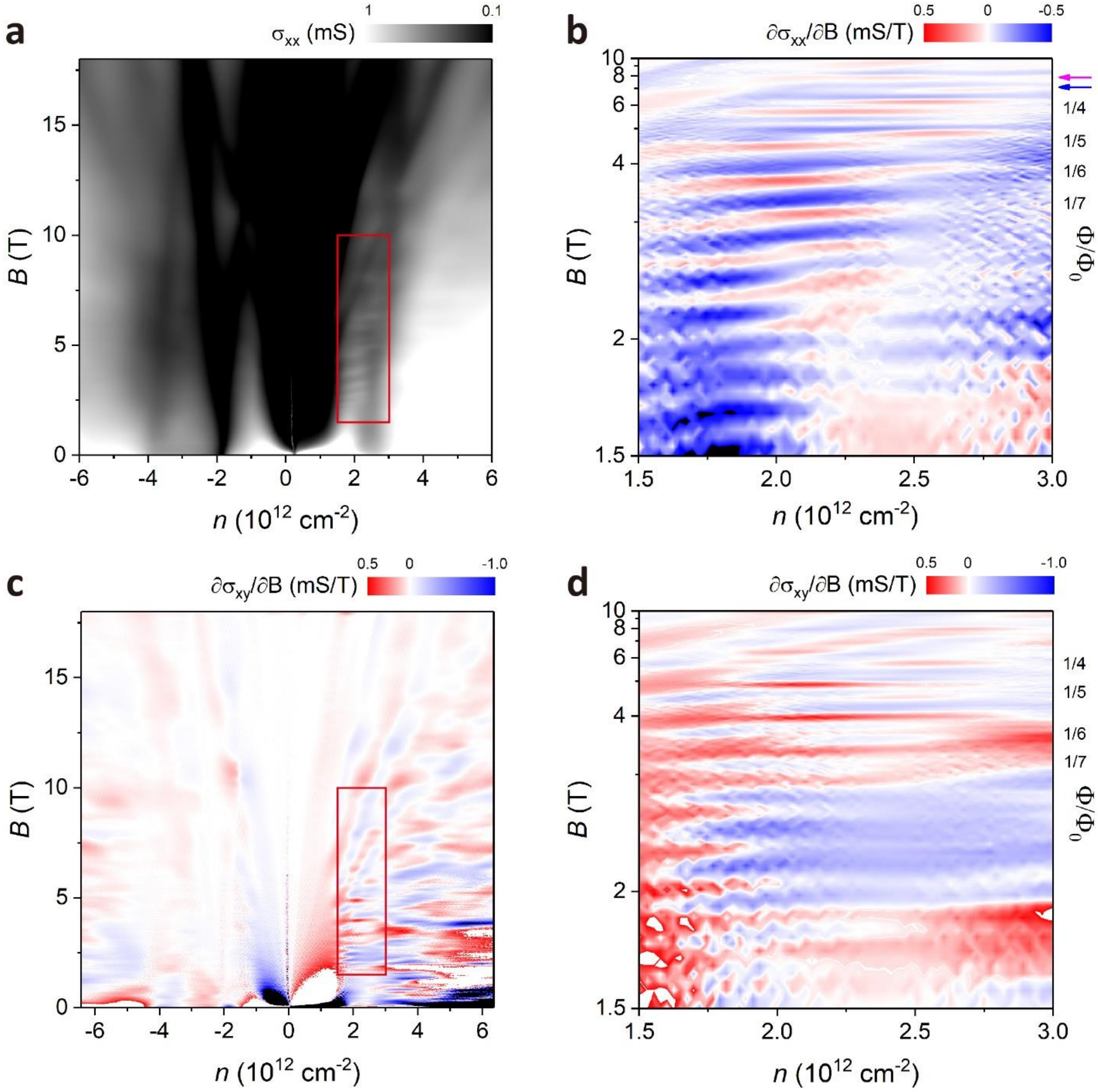

Fig. S13. Brown-Zak oscillations in double moiré monolayer graphene at T = 70 K. (a) Longitudinal conductivity σ_{xx} as a function of n and B, $\sigma_{xx}(n,B)$. (b) $\partial\sigma_{xx}/\partial B(n,B)$ in part of (a) near the SDPs for electron doping (marked in (a) by a red rectangle). The blue and magenta arrows show the Brown-Zak oscillations belonging to the SDPs at $n_{s1} = \pm 2.10\times10^{12}$ cm^{-2}, and $n_{s2} = \pm 2.44\times10^{12}$ cm^{-2}, respectively. (c) $\partial\sigma_{xy}/\partial B(n,B)$. (d) Part of (c) near the SDPs for electron doping (marked in (c) by a red rectangle). The numbers on the right in (b) and (d) indicate $\phi = (1/q)\ \phi_0$ with q = 4 to 7.

Supplementary Movies

Supplementary Movie S1. Rotation of van der Waals heterostructure. The movie shows the rotation of top hBN and graphene in an hBN/graphene/hBN stack under the control of PMMA patch deposited on top of the top hBN. The PMMA patch detached from top hBN after the clicking between top hBN, graphene and bottom hBN.

Supplementary Movie S2. Displacement of van der Waals heterostructure. The movie shows the displacement of top hBN and graphene in the same hBN/graphene/hBN stack under the control of PMMA patch deposited on top of the top hBN.

REFERENCES AND NOTES

1. Y.-H. Zhang, D. Mao, Y. Cao, P. Jarillo-Herrero, T. Senthil, Nearly flat Chern bands in moiré superlattices. *Phys. Rev. B* **99**, 075127 (2019).

2. S. Carr, D. Massatt, S. Fang, P. Cazeaux, M. Luskin, E. Kaxiras, Twistronics: Manipulating the electronic properties of two-dimensional layered structures through their twist angle. *Phys. Rev. B* **95**, 075420 (2017).

3. F. Wu, T. Lovorn, E. Tutuc, I. Martin, A. H. MacDonald, Topological insulators in twisted transition metal dichalcogenide homobilayers. *Phys. Rev. Lett.* **122**, 086402 (2019).

4. X. Chen, J. R. Wallbank, A. A. Patel, M. Mucha-Kruczyński, E. McCann, V. I. Fal'ko, Dirac edges of fractal magnetic minibands in graphene with hexagonal moiré superlattices. *Phys. Rev. B* **89**, 075401 (2014).

5. L. A. Ponomarenko, R. V. Gorbachev, G. L. Yu, D. C. Elias, R. Jalil, A. A. Patel, A. Mishchenko, A. S. Mayorov, C. R. Woods, J. R. Wallbank, M. Mucha-Kruczynski, B. A. Piot, M. Potemski, I. V. Grigorieva, K. S. Novoselov, F. Guinea, V. I. Fal'ko, A. K. Geim, Cloning of Dirac fermions in graphene superlattices. *Nature* **497**, 594–597 (2013).

6. C. R. Dean, L. Wang, P. Maher, C. Forsythe, F. Ghahari, Y. Gao, J. Katoch, M. Ishigami, P. Moon, M. Koshino, T. Taniguchi, K. Watanabe, K. L. Shepard, J. Hone, P. Kim, Hofstadter's butterfly and the fractal quantum Hall effect in moiré superlattices. *Nature* **497**, 598–602 (2013).

7. B. Hunt, J. D. Sanchez-Yamagishi, A. F. Young, M. Yankowitz, B. J. LeRoy, K. Watanabe, T. Taniguchi, P. Moon, M. Koshino, P. Jarillo-Herrero, R. C. Ashoori, Massive Dirac fermions and Hofstadter butterfly in a van der Waals heterostructure. *Science* **340**, 1427–1430 (2013).

8. G. Chen, L. Jiang, S. Wu, B. Lyu, H. Li, B. L. Chittari, K. Watanabe, T. Taniguchi, Z. Shi, J. Jung, Y. Zhang, F. Wang, Evidence of a gate-tunable Mott insulator in a trilayer graphene moiré superlattice. *Nat. Phys.* **15**, 237–241 (2019).

9. G. Chen, A. L. Sharpe, P. Gallagher, I. T. Rosen, E. J. Fox, L. Jiang, B. Lyu, H. Li, K. Watanabe, T. Taniguchi, J. Jung, Z. Shi, D. Goldhaber-Gordon, Y. Zhang, F. Wang, Signatures of tunable superconductivity in a trilayer graphene moiré superlattice. *Nature* **572**, 215–219 (2019).

10. E. M. Spanton, A. A. Zibrov, H. Zhou, T. Taniguchi, K. Watanabe, M. P. Zaletel, A. F. Young, Observation of fractional Chern insulators in a van der Waals heterostructure. *Science* **360**, 62–66 (2018).

11. A. L. Sharpe, E. J. Fox, A. W. Barnard, J. Finney, K. Watanabe, T. Taniguchi, M. A. Kastner, D. Goldhaber-Gordon, Emergent ferromagnetism near three-quarters filling in twisted bilayer graphene. *Science* **365**, 605–608 (2019).

12. Y. Cao, V. Fatemi, A. Demir, S. Fang, S. L. Tomarken, J. Y. Luo, J. D. Sanchez-Yamagishi, K. Watanabe, T. Taniguchi, E. Kaxiras, R. C. Ashoori, P. Jarillo-Herrero, Correlated insulator behaviour at half-filling in magic-angle graphene superlattices. *Nature* **556**, 80–84 (2018).

13. Y. Cao, V. Fatemi, S. Fang, K. Watanabe, T. Taniguchi, E. Kaxiras, P. Jarillo-Herrero, Unconventional superconductivity in magic-angle graphene superlattices. *Nature* **556**, 43–50 (2018).

14. M. Yankowitz, S. Chen, H. Polshyn, Y. Zhang, K. Watanabe, T. Taniguchi, D. Graf, A. F. Young, C. R. Dean, Tuning superconductivity in twisted bilayer graphene. *Science* **363**, 1059–1064 (2019).

15. C. Shen, Y. Chu, Q. Wu, N. Li, S. Wang, Y. Zhao, J. Tang, J. Liu, J. Tian, K. Watanabe, T. Taniguchi, R. Yang, Z. Y. Meng, D. Shi, O. V. Yazyev, G. Zhang, Correlated states in twisted double bilayer graphene. *Nat. Phys.* **16**, 520–525 (2020).

16. A. Kerelsky, C. Rubio-Verdú, L. Xian, D. M. Kennes, D. Halbertal, N. Finney, L. Song, S. Turkel, L. Wang, K. Watanabe, T. Taniguchi, J. Hone, C. Dean, D. Basov, A. Rubio, A. N. Pasupathy, Moiré-less correlations in ABCA graphene. *arXiv:1911.00007* (2019).

17. X. Liu, Z. Hao, E. Khalaf, J. Y. Lee, Y. Ronen, H. Yoo, D. Haei Najafabadi, K. Watanabe, T. Taniguchi, A. Vishwanath, P. Kim, Tunable spin-polarized correlated states in twisted double bilayer graphene. *Nature* **583**, 221–225 (2020).

18. Y. Cao, D. Rodan-Legrain, O. Rubies-Bigorda, J. M. Park, K. Watanabe, T. Taniguchi, P. Jarillo-Herrero, Tunable correlated states and spin-polarized phases in twisted bilayer-bilayer graphene. *Nature* **583**, 215–220 (2020).

19. E. M. Alexeev, D. A. Ruiz-Tijerina, M. Danovich, M. J. Hamer, D. J. Terry, P. K. Nayak, S. Ahn, S. Pak, J. Lee, J. I. Sohn, M. R. Molas, M. Koperski, K. Watanabe, T. Taniguchi, K. S. Novoselov, R. V. Gorbachev, H. S. Shin, V. I. Fal'ko, A. I. Tartakovskii, Resonantly hybridized excitons in moiré superlattices in van der Waals heterostructures. *Nature* **567**, 81–86 (2019).

20. K. L. Seyler, P. Rivera, H. Yu, N. P. Wilson, E. L. Ray, D. G. Mandrus, J. Yan, W. Yao, X. Xu, Signatures of moiré-trapped valley excitons in $MoSe_2$/WSe_2 heterobilayers. *Nature* **567**, 66–70 (2019).

21. L. Wang, E.-M. Shih, A. Ghiotto, L. Xian, D. A. Rhodes, C. Tan, M. Claassen, D. M. Kennes, Y. Bai, B. Kim, K. Watanabe, T. Taniguchi, X. Zhu, J. Hone, A. Rubio, A. N. Pasupathy, C. R. Dean, Correlated electronic phases in twisted bilayer transition metal dichalcogenides. *Nat. Mater.* **19**, 861–866 (2020).

22. L. An, X. Cai, D. Pei, M. Huang, Z. Wu, Z. Zhou, J. Lin, Z. Ying, Z. Ye, X. Feng, R. Gao, C. Cacho, M. Watson, Y. Chen, N. Wang, Interaction effects and superconductivity signatures in twisted double-bilayer WSe_2. *Nanoscale Horiz.* **5**, 1309–1316 (2020).

23. S. Ulstrup, R. J. Koch, S. Singh, K. M. McCreary, B. T. Jonker, J. T. Robinson, C. Jozwiak, E. Rotenberg, A. Bostwick, J. Katoch, J. A. Miwa, Direct observation of minibands in a twisted graphene/WS_2 bilayer. *Sci. Adv.* **6**, eaay6104 (2020).

24. M. Serlin, C. L. Tschirhart, H. Polshyn, Y. Zhang, J. Zhu, K. Watanabe, T. Taniguchi, L. Balents, A. F. Young, Intrinsic quantized anomalous Hall effect in a moiré heterostructure. *Science* **367**, 900–903 (2020).

25. C. R. Woods, L. Britnell, A. Eckmann, R. S. Ma, J. C. Lu, H. M. Guo, X. Lin, G. L. Yu, Y. Cao, R. V. Gorbachev, A. V. Kretinin, J. Park, L. A. Ponomarenko, M. I. Katsnelson, Y. N. Gornostyrev, K. Watanabe, T. Taniguchi, C. Casiraghi, H.-J. Gao, A. K. Geim, K. S. Novoselov, Commensurate–incommensurate transition in graphene on hexagonal boron nitride. *Nat. Phys.* **10**, 451–456 (2014).

26. K. Kim, M. Yankowitz, B. Fallahazad, S. Kang, H. C. P. Movva, S. Huang, S. Larentis, C. M. Corbet, T. Taniguchi, K. Watanabe, S. K. Banerjee, B. J. LeRoy, E. Tutuc, van der Waals heterostructures with high accuracy rotational alignment. *Nano Lett.* **16**, 1989–1995 (2016).

27. R. Ribeiro-Palau, C. Zhang, K. Watanabe, T. Taniguchi, J. Hone, C. R. Dean, Twistable electronics with dynamically rotatable heterostructures. *Science* **361**, 690–693 (2018).

28. Y. Wakafuji, R. Moriya, S. Masubuchi, K. Watanabe, T. Taniguchi, T. Machida, 3D manipulation of 2D materials using microdome polymer. *Nano Lett.* **20**, 2486–2492 (2020).

29. X. Feng, S. Kwon, J. Y. Park, M. Salmeron, Superlubric sliding of graphene nanoflakes on graphene. *ACS Nano* **7**, 1718–1724 (2013).

30. A. Vanossi, N. Manini, M. Urbakh, S. Zapperi, E. Tosatti, *Colloquium*: Modeling friction: From nanoscale to mesoscale. *Rev. Mod. Phys.* **85**, 529–552 (2013).

31. Z. Liu, J. Yang, F. Grey, J. Z. Liu, Y. Liu, Y. Wang, Y. Yang, Y. Cheng, Q. Zheng, Observation of microscale superlubricity in graphite. *Phys. Rev. Lett.* **108**, 205503 (2012).

32. E. Koren, U. Duerig, Moiré scaling of the sliding force in twisted bilayer graphene. *Phys. Rev. B* **94**, 045401 (2016).

33. K. Yao, N. R. Finney, J. Zhang, S. L. Moore, L. Xian, N. Tancogne-Dejean, F. Liu, J. Ardelean, X. Xu, D. Halbertal, K. Watanabe, T. Taniguchi, H. Ochoa, A. Asenjo-Garcia, X. Zhu, D. N. Basov, A. Rubio, C. R. Dean, J. Hone, P. J. Schuck, Nonlinear twistoptics at symmetry-broken interfaces. *arXiv:2006.13802* (2020).

34. N. R. Finney, M. Yankowitz, L. Muraleetharan, K. Watanabe, T. Taniguchi, C. R. Dean, J. Hone, Tunable crystal symmetry in graphene-boron nitride heterostructures with coexisting moiré superlattices. *Nat. Nanotechnol.* **14**, 1029–1034 (2019).

35. A. Eckmann, J. Park, H. Yang, D. Elias, A. S. Mayorov, G. Yu, R. Jalil, K. S. Novoselov, R. V. Gorbachev, M. Lazzeri, A. K. Geim, C. Casiraghi, Raman fingerprint of aligned graphene/h-BN superlattices. *Nano Lett.* **13**, 5242–5246 (2013).

36. A. H. MacDonald, Landau-level subband structure of electrons on a square lattice. *Phys. Rev. B* **28**, 6713–6717 (1983).

37. R. Krishna Kumar, X. Chen, G. H. Auton, A. Mishchenko, D. A. Bandurin, S. V. Morozov, Y. Cao, E. Khestanova, M. Ben Shalom, A. V. Kretinin, K. S. Novoselov, L. Eaves, I. V. Grigorieva, L. A. Ponomarenko, V. I. Fal'ko, A. K. Geim, High-temperature quantum oscillations caused by recurring Bloch states in graphene superlattices. *Science* **357**, 181–184 (2017).

38. L. Wang, S. Zihlmann, M.-H. Liu, P. Makk, K. Watanabe, T. Taniguchi, A. Baumgartner, C. Schönenberger, New generation of moiré superlattices in doubly aligned hBN/graphene/hBN heterostructures. *Nano Lett.* **19**, 2371–2376 (2019).

39. Z. Wang, Y. B. Wang, J. Yin, E. Tóvári, Y. Yang, L. Lin, M. Holwill, J. Birkbeck, D. J. Perello, S. Xu, J. Zultak, R. V. Gorbachev, A. V. Kretinin, T. Taniguchi, K. Watanabe, S. V. Morozov, M. Anđelković, S. P. Milovanović, L. Covaci, F. M. Peeters, A. Mishchenko, A. K. Geim, K. S. Novoselov, V. I. Fal'ko, A. Knothe, C. R. Woods, Composite super-moiré lattices in double-aligned graphene heterostructures. *Sci. Adv.* **5**, eaay8897 (2019).

40. M. Onodera, K. Kinoshita, R. Moriya, S. Masubuchi, K. Watanabe, T. Taniguchi, T. Machida, Cyclotron resonance study of monolayer graphene under double moiré potentials. *Nano Lett.* **20**, 4566–4572 (2020).

41. N. Leconte, J. Jung, Commensurate and incommensurate double moire interference in graphene encapsulated by hexagonal boron nitride. *2D Mater.* **7**, 031005 (2020).

42. M. Andelkovic, S. P. Milovanovic, L. Covaci, F. M. Peeters, Double Moiré with a twist: Supermoiré in encapsulated graphene. *Nano Lett.* **20**, 979–988 (2019).

43. S. J. Ahn, P. Moon, T. H. Kim, H. W. Kim, H. C. Shin, E. H. Kim, H. W. Cha, S. J. Kahng, P. Kim, M. Koshino, Y. W. Son, C. W. Yang, J. R. Ahn, Dirac electrons in a dodecagonal graphene quasicrystal. *Science* **361**, 782–786 (2018).

44. P. Moon, M. Koshino, Y.-W. Son, Quasicrystalline electronic states in 30° rotated twisted bilayer graphene. *Phys. Rev. B* **99**, 165430 (2019).

45. S. G. Xu, A. I. Berdyugin, P. Kumaravadivel, F. Guinea, R. Krishna Kumar, D. A. Bandurin, S. V. Morozov, W. Kuang, B. Tsim, S. Liu, J. H. Edgar, I. V. Grigorieva, V. I. Fal'ko, M. Kim, A. K. Geim, Giant oscillations in a triangular network of one-dimensional states in marginally twisted graphene. *Nat. Commun.* **10**, 4008 (2019).

46. T. Uwanno, Y. Hattori, T. Taniguchi, K. Watanabe, K. Nagashio, Fully dry PMMA transfer of graphene onh-BN using a heating/cooling system. *2D Mater.* **2**, 041002 (2015).

47. M. Yankowitz, J. Xue, D. Cormode, J. D. Sanchez-Yamagishi, K. Watanabe, T. Taniguchi, P. Jarillo-Herrero, P. Jacquod, B. J. LeRoy, Emergence of superlattice Dirac points in graphene on hexagonal boron nitride. *Nat. Phys.* **8**, 382–386 (2012).

48. Y. Song, D. Mandelli, O. Hod, M. Urbakh, M. Ma, Q. Zheng, Robust microscale superlubricity in graphite/hexagonal boron nitride layered heterojunctions. *Nat. Mater.* **17**, 894–899 (2018).

49. B. Cheng, P. Wang, C. Pan, T. Miao, Y. Wu, T. Taniguchi, K. Watanabe, C. N. Lau, M. Bockrath, Raman spectroscopy measurement of bilayer graphene's twist angle to boron nitride. *Appl. Phys. Lett.* **107**, 033101 (2015).

50. M. Huang, H. Yan, C. Chen, D. Song, T. F. Heinz, J. Hone, Phonon softening and crystallographic orientation of strained graphene studied by Raman spectroscopy. *Proc. Natl. Acad. Sci. U.S.A.* **106**, 7304–7308 (2009).

51. T. M. G. Mohiuddin, A. Lombardo, R. R. Nair, A. Bonetti, G. Savini, R. Jalil, N. Bonini, D. M. Basko, C. Galiotis, N. Marzari, K. S. Novoselov, A. K. Geim, A. C. Ferrari, Uniaxial strain in graphene by Raman spectroscopy: Gpeak splitting, Grüneisen parameters, and sample orientation. *Phys. Rev. B* **79**, 205433 (2009).

52. L. J. McGilly, A. Kerelsky, N. R. Finney, K. Shapovalov, E. M. Shih, A. Ghiotto, Y. Zeng, S. L. Moore, W. Wu, Y. Bai, K. Watanabe, T. Taniguchi, M. Stengel, L. Zhou, J. Hone, X. Zhu, D. N. Basov, C. Dean, C. E. Dreyer, A. N. Pasupathy, Visualization of moiré superlattices. *Nat. Nanotechnol.* **15**, 580–584 (2020).

53. A. Das, S. Pisana, B. Chakraborty, S. Piscanec, S. K. Saha, U. V. Waghmare, K. S. Novoselov, H. R. Krishnamurthy, A. K. Geim, A. C. Ferrari, A. K. Sood, Monitoring dopants by Raman scattering in an electrochemically top-gated graphene transistor. *Nat. Nanotechnol.* **3**, 210–215 (2008).

54. S. Pisana, M. Lazzeri, C. Casiraghi, K. S. Novoselov, A. K. Geim, A. C. Ferrari, F. Mauri, Breakdown of the adiabatic Born-Oppenheimer approximation in graphene. *Nat. Mater.* **6**, 198–201 (2007).

55. J. R. Wallbank, A. A. Patel, M. Mucha-Kruczyński, A. K. Geim, V. I. Fal'ko, Generic miniband structure of graphene on a hexagonal substrate. *Phys. Rev. B* **87**, 245408 (2013).
